# Understanding of Adventitious Root Formation: What Can We Learn From Comparative Genetics?

**DOI:** 10.3389/fpls.2020.582020

**Published:** 2020-10-06

**Authors:** Mariem Mhimdi, José Manuel Pérez-Pérez

**Affiliations:** Instituto de Bioingeniería, Universidad Miguel Hernández, Alicante, Spain

**Keywords:** crown roots, adventitious rooting, waterlogging stress, hormone crosstalk, polar auxin transport, reactive oxygen species, microRNA regulation

## Abstract

Adventitious root (AR) formation is a complex developmental process controlled by a plethora of endogenous and environmental factors. Based on fossil evidence and genomic phylogeny, AR formation might be considered the default state of plant roots, which likely evolved independently several times. The application of next-generation sequencing techniques and bioinformatics analyses to non-model plants provide novel approaches to identify genes putatively involved in AR formation in multiple species. Recent results uncovered that the regulation of shoot-borne AR formation in monocots is an adaptive response to nutrient and water deficiency that enhances topsoil foraging and improves plant performance. A hierarchy of transcription factors required for AR initiation has been identified from genetic studies, and recent results highlighted the key involvement of additional regulation through microRNAs. Here, we discuss our current understanding of AR formation in response to specific environmental stresses, such as nutrient deficiency, drought or waterlogging, aimed at providing evidence for the integration of the hormone crosstalk required for the activation of root competent cells within adult tissues from which the ARs develop.

## Introduction

By definition, adventitious roots (AR) originate post-embryonically from tissues other than roots in response to different environmental signals, in a process that is highly regulated by hormonal crosstalk (Atkinson et al., 2014; Bellini et al., 2014; Steffens and Rasmussen, 2016; Gonin et al., 2019; Lakehal and Bellini, 2019). During normal development, many plant species develop ARs to perform specialized functions, such as increasing soil exploration (e.g., crown roots in monocots), providing support for aerial organs (e.g., pillar roots in *Ficus* spp. and mangroves, crown and brace roots in maize), enhancing water capture (e.g., aerial roots in epiphyte plants), or allowing vegetative propagation of buried stems (e.g., runners in *Fragaria vesca*). In many dicots, however, the postembryonic root system is mainly composed of lateral roots (LRs), which originate from the pericycle cell layer of already formed roots (Dubrovsky et al., 2006; Péret et al., 2009). In these species, ARs are also formed, mainly in response to specific stress signals, such as waterlogging and wounding, the latter being usually applied during stem cutting propagation (Druege et al., 2019).

## An Ancient Evolutionary Origin of ARs

Based on the fossil evidence and the root anatomy of extant vascular plants, two separate root-evolution events in Lycophytes (such as *Selaginella moellendorffii*) and Euphyllophytes (ferns and seed plants) have been proposed (Pires and Dolan, 2012). Rhizoid-based rooting systems in lycophytes that preceded the evolution of true roots in early land plants likely developed from dormant meristematic regions on aerial axes when in proximity to the soil, and contributed to water and nutrient absorption, as in modern bryophytes (Hetherington and Dolan, 2018). The large variation in root morphology among extant lycophytes supports the hypothesis of multiple evolution events of roots in the lycophyte lineage (Fujinami et al., 2017).

True roots also independently appeared in several clades during the Devonian Period as forest ecosystems evolved (Kenrick and Strullu-Derrien, 2014). Approximately 385 million years ago, the extinct Cladoxylales, thought to be intermediate between early vascular plants and living ferns, were anchored at their trunk base by many narrow, unbranched, overlapping ARs, similar to modern palms (Kenrick and Strullu-Derrien, 2014). Similar to lycophytes, extant ferns usually have a homorhizic root system (all roots originate from the shoot system and are, by definition, shoot-borne ARs), while seed plants have primary roots (PRs) with LRs (i.e., allorhizic root system), which has been interpreted as a third root-evolutionary event (Liu and Xu, 2018). However, it is currently unknown whether the allorhizic seed plant root is homologous with the homorhizic fern root (Augstein and Carlsbecker, 2018).

In vascular plants, *WUSCHEL RELATED HOMEOBOX* (*WOX*) genes are associated with stem cell regulation (Dolzblasz et al., 2016). *WOX* genes from the intermediate clade (IC-*WOX* genes) displayed evolutionary conserved functions in root organogenesis (Liu and Xu, 2018). In the fern *Ceratopteris richardii*, an IC-*WOX* gene, *CrWOXA*, was specifically expressed in root founder cells during LR and AR initiation, which led to the hypothesis that IC-*WOX* genes were recruited in the common ancestor of ferns and seed plants for AR organogenesis and later evolved into two subclades in seed plants: one was retained in AR organogenesis (*WOX11/12*), while the other was recruited for PR specification during embryogenesis (*WOX8/9*) (Liu and Xu, 2018). Additional experiments have demonstrated that *CrWOXA* could be a direct target of the auxin signaling pathway, which is sufficient to trigger AR initiation in *C. richardii* (Yu et al., 2020). In turn, *CrWOXA* might directly activate the WUSCHEL-clade *WOX* (WC-*WOX*) gene, *CrWUL*, similar to the direct activation of *WOX5* expression by WOX11 required for the root founder cell division that forms the root primordium during AR initiation in *Arabidopsis thaliana* (hereafter Arabidopsis) leaf explants (Hu and Xu, 2016). On the other hand, ectopic expression of *CrWOXA* in Arabidopsis leaf explants enhanced adventitious rooting (Yu et al., 2020). These results are in agreement with a conserved role of IC-*WOX* genes in the regulation of AR initiation in Euphyllophytes in two steps: (i) establishing the root founder cell downstream of the auxin signal and (ii) positioning the stem cell niche within the newly formed root primordia.

**FIGURE 1:**
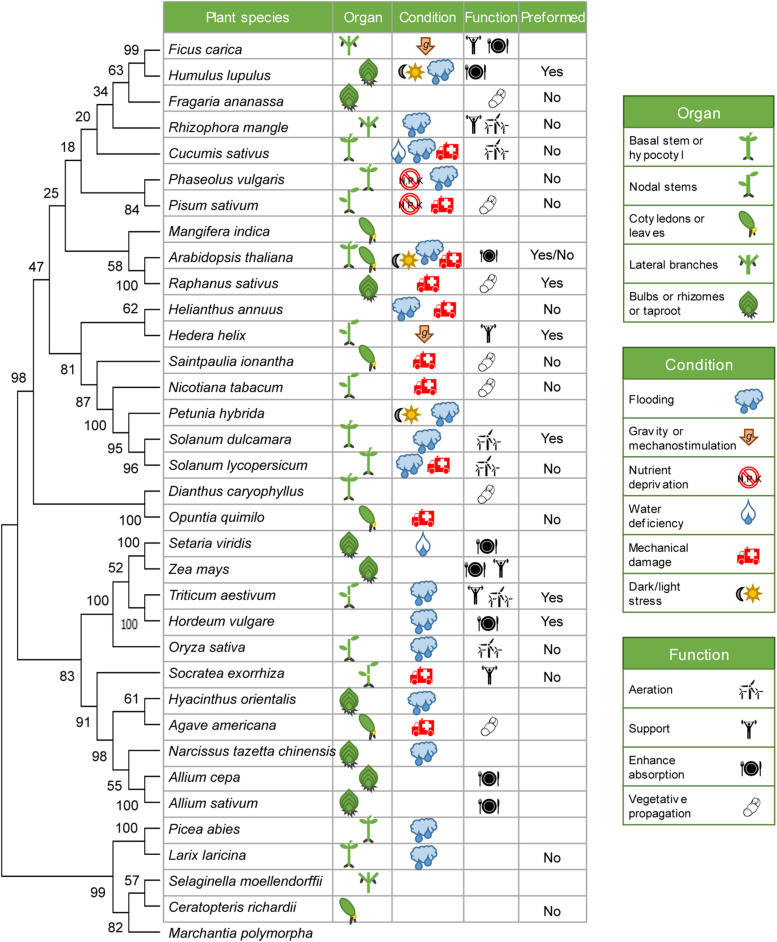
Evolutionary constraints of AR formation. *rbcL* nucleotide sequences from 34 representative plant species where AR formation has been experimentally described were retrieved from NCBI (Supplementary Dataset) and aligned by the ClustalW algorithm with MEGA-X (Kumar et al., 2018). The liverwort *Marchantia polymorpha* has been used here as an outgroup. The consensus phylogenetic tree was inferred by using the maximum likelihood (ML) method and Jones-Taylor-Thornton (JTT) model from 500 bootstrap replicates. The percentage of replicate trees in which the associated taxa clustered together in the bootstrap test are shown next to the branches. Some details of their AR system of each species are provided next to the species name.

We built an evolutionary consensus tree including 34 representative plant species in which the AR system has been studied in some detail (Figure 1). In most of them, ARs were induced in the basal region of stem cuttings during vegetative propagation, normally triggered by exogenous auxin treatment. However, in some species, ARs are normally repressed in aerial tissues but might be locally induced by stress signals, mainly waterlogging, nutrient deficiency and mechanical damage (Figure 1). ARs are also formed *de novo* from vascular cells in adult leaves or stems in dicots after wounding (Figure 1). In any event, root initiation from non-root (shoot-borne) tissues might be considered as an ancestral trait, as ARs are formed by default in lycophytes (*S. moellendorfii*) and ferns (*C. richardii*), while AR formation in many angiosperms might be repressed through unknown mechanism(s) in the absence of the specific stress signal.

## Genetic Variation of Excision-Induced AR Formation

We previously discussed about the contribution of genetic variation approaches for the molecular understanding of excision-induced AR formation in stem cuttings of important woody species such as poplar, *Eucalyptus* spp., and olive trees (Druege et al., 2019). In Arabidopsis, excised leaf explants are able to produce ARs from some vascular associated cells (Liu et al., 2014; Bustillo-Avendaño et al., 2018), and recent genetic studies have identified several key regulators that are involved in this process (Jing et al., 2020). Although many of these regulators also function during LR development, others, such as WOX11, are AR-specific (Jing et al., 2020). In a recent study, an integrative analysis of quantitative trait loci (QTL) mapping and RNA-Seq data on a full-sib F_1_ population from a cross between female *Populus deltoides* “Danhong” and male *Populus simonii* “Tongliao1” resulted in the identification of 14 QTL clusters and hundreds of candidate differentially expressed genes affecting several excision-induced AR traits (Sun et al., 2019). One of the candidate genes identified, *PtAAAP19*, encoded a transmembrane protein putatively involved in amino acid uptake in roots (Sun et al., 2019). In another study in *Catalpa* spp., association mapping for three adventitious rooting traits was performed on a selected germplasm collection, which resulted in the identification of a gene, *CbNN1*, with high expression levels in accessions with greater adventitious rooting ability (Wang P. et al., 2019). *CbNN1* encodes a WRKY transcription factor, whose Arabidopsis ortholog modulates the crosstalk between the salicylic acid and jasmonic acid (JA) pathways in response to a wide range of biotic and abiotic stimuli (Kloth et al., 2016). None of these two genes was previously linked to excision-induced AR formation. Although next-generation sequencing techniques and bioinformatics analysis provide novel genes putatively involved in excision-induced AR formation in different species, their explicit role in AR formation still requires experimental validation.

## Role of Shoot-Borne Root Architecture in Nutrient Capture and Water Availability

Monocots develop a complex root system composed of both embryonic roots and postembryonic roots, which are named according to the tissue of origin as seminal roots and shoot-borne crown roots (CRs); in some species such as maize (*Zea mays*), brace roots are also produced from higher internodes at the stem (Martínez-de la Cruz et al., 2015; Hochholdinger et al., 2018). CRs play important roles in anchorage and soil resource acquisition during vegetative growth and reproductive development (Hochholdinger, 2016). All types of roots produce secondary roots (i.e., LRs) that originate from the pericycle of already existing roots. Several reports highlighted the transcriptomic, anatomical, and physiological complexity of the different types of roots produced in rice (Takehisa et al., 2012; Gutjahr et al., 2015) and maize (Zhang et al., 2015; Tai et al., 2016; Yu et al., 2016).

**FIGURE 2:**
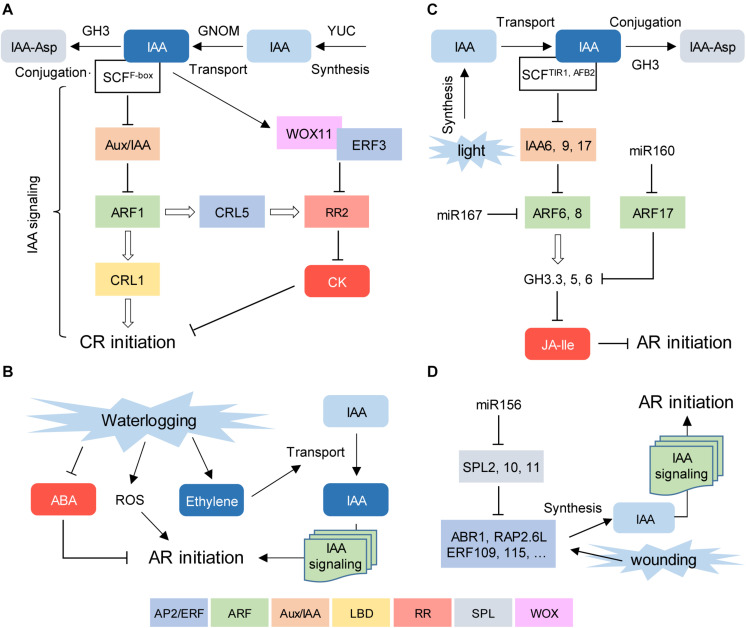
Conserved regulatory pathways for AR formation. **(A)** Early steps during crown root (CR) formation in rice seedlings. **(B)** Waterlogging-induced AR formation in bittersweet. **(C)** Light-stress induced AR formation in *Arabidopsis thaliana* hypocotyls. **(D)** Proposed role of miR156 regulation in wound-induced AR formation in *A. thaliana* leaf explants. Positive and negative hormonal regulators are shown in blue and red, respectively. Transcriptional regulators are depicted inside boxes of different colors. Each color represents a given DNA binding domain (see main text for legends).

In rice, both LR and CR formation is controlled by *GNOM*, which encodes a guanine-nucleotide exchange factor involved in polar auxin transport regulation (Liu et al., 2009). Downstream of auxin, CR formation is positively regulated by CROWN ROOTLESS1/ADVENTITIOUS ROOTLESS1 (CRL1/ARL1) and WOX11. CRL1 belongs to the LATERAL ORGAN BOUNDARIES domain (LBD) transcription factor family and regulates CR formation downstream of AUXIN RESPONSE FACTOR1 (ARF1; Inukai et al., 2005; Liu et al., 2005). WOX11 functions in CR initiation by directly repressing the expression of the type-A negative regulator of cytokinin signaling *RESPONSE REGULATOR2* (*OsRR2*; Zhao et al., 2009) in cooperation with ERF3, an APETALA2/ETHYLENE RESPONSE FACTOR (AP2/ERF) transcription factor (Zhao et al., 2015). CRL5, another AP2/ERF transcription factor, upregulates *OsRR1* and *OsRR2* expression independently of CRL1 (Kitomi et al., 2011). Hence, CR initiation in rice relies on a direct crosstalk between auxin and cytokinin signaling through concerted transcription factor regulation (Figure 2A).

In maize, shoot-borne CR initiation is controlled by *ROOTLESS CONCERNING CROWN AND SEMINAL ROOTS* (*RCTS*), which encodes an auxin-inducible LBD transcription factor (Taramino et al., 2007). *RCTS* and its paralog *RCTL* cooperatively act downstream of the auxin response factor ARF34 during CR development (Xu et al., 2015). CR number significantly varied among maize genotypes in a quantitative manner (Burton et al., 2014) and it has been suggested that low CR numbers improve resource acquisition under drought (Gao and Lynch, 2016). On the other hand, a recent study found non-significant genotype × environment (G × E) interaction between CR number and different water regimes in a recombinant inbred line (RIL) population of maize (Li et al., 2018). Several QTLs associated with CR angle and CR length were identified instead as having antagonistic pleiotropic effects in response to water deficiency, a result that might be linked to the partially overlapping function of RCTS and RCTL (Li et al., 2018). Conversely, maize genotypes with large CR number have shallower rooting depth and improved phosphate acquisition from low-phosphate soils (Sun et al., 2018). These results indicate that topsoil foraging helps to improve plant performance under suboptimal nutrient availability.

In the C_4_ model grass *Setaria viridis*, suppression of CR growth was a major response to water deficiency. Unlike *S. viridis*, cultivated foxtail millet, *S. italica*, maintained the ability to produce a small number of CR under water deficiency conditions (Sebastian et al., 2016). These results suggested that water availability was locally sensed by the basal stem of *S. viridis* to directly regulate CR initiation. To identify the genetic factors involved in these differential response to water deficiency, a QTL analysis using a recombinant inbred line (RIL) population from a cross of *S. viridis*, and *S. italica* was performed. As a result, two major QTLs for CR variation in response to water availability were identified; of the 380 genes in the confidence interval of one of these QTLs, 28 were differentially expressed in the CRs under water deficit conditions, thus identifying potential candidates for further study (Sebastian et al., 2016). Interestingly, the water deficiency-induced CR inhibition observed in *S. viridis* was confirmed in other Poaceae species: sorghum (*Sorghum bicolor*), switch grass (*Panicum virgatum*), and *Brachypodium distachyon* (Sebastian et al., 2016). Based on these results, the authors proposed that the transition from a PR-dominated system to a CR-dominated system might be a key adaptation that allows grasses to rapidly increase root growth in response to recent precipitation events. CRs of *Zea mays* and *Setaria italica*, domesticated relatives of teosinte and *S. viridis*, respectively, showed reduced sensitivity to water deficit conditions, suggesting that this response might have been influenced by human selection (Sebastian et al., 2016). On the other hand, sorghum, which completely abolishes CR growth under water deficiency, exhibit significant drought tolerance for a crop species. Targeted breeding approaches for varieties with enhanced water deficiency responses of CRs in other crops may increase yield when water availability is compromised.

## Conserved Hormonal Pathways During Waterlogging-Induced AR Emergence

An adaptive response of many species to waterlogging stress is the emergence of ARs, which facilitate gas transport and nutrient uptake. AR primordia can be formed during normal development and emerge upon flooding, or they can develop *de novo* in response to flooding (Steffens and Rasmussen, 2016). Bittersweet (*Solanum dulcamara*), which is closely related to waterlogging-intolerant tomato and eggplant crops, has been proposed as a model to study waterlogging-induced AR emergence (Dawood et al., 2014). The activation of preformed AR primordia in the stem initiates within 24 h of partial submergence, but the molecular responses begin within 2 h and include activation of hypoxia-responsive and ethylene signaling genes (Dawood et al., 2014). Downstream of ethylene, the reduction in abscisic acid (ABA) levels in dormant AR primordia followed the reactivation of cell divisions which were directly dependent on the auxin that was continuously produced in the shoot and actively transported through the stem (Dawood et al., 2016; Yang et al., 2018; Figure 2B).

A similar hormone crosstalk induces growth of preformed ARs (e.g., nodal roots) in deep-water rice when plants become submerged (Steffens et al., 2006). In non-flooded conditions, light inhibited AR emergence through an unknown mechanism. Upon flooding, the growth of the activated AR primordia within the vasculature induces reactive oxygen species (ROS)-dependent cell death of the overlying epidermis through local mechanical sensing (Steffens et al., 2012). In these two examples, the hormone crosstalk required for AR emergence upon flooding derived from the redeployment of pre-existing developmental and signaling pathways controlling seedling germination and shoot branching. Hence, it is tempting to speculate that orthologs of some of the genes involved in regulating seed and bud dormancy, such as *ABSCISIC ACID INSENSITIVE5* (Skubacz et al., 2016) and *BRANCHED1* (Wang M. et al., 2019) might also contribute to AR primordia dormancy in these two species during flooding-induced AR emergence.

In wheat (*Triticum aestivum*), waterlogging induced the formation of nodal roots with aerenchyma tissues, a response that was associated with enhanced expression of some ethylene biosynthetic genes and *RESPIRATORY BURST OXIDASE HOMOLOG* (*RBOH*) genes in the submerged stem (Nguyen et al., 2018). Indeed, ethylene and ROS production also promote emergence of nodal roots in wheat under low-oxygen conditions (Yamauchi et al., 2014). In addition, enhanced expression of auxin biosynthesis and transport genes in waterlogged stems directly contributed to higher auxin levels required for nodal root induction (Nguyen et al., 2018). These results highlight the importance of auxin–ethylene interplay in regulating the formation of nodal roots from stem nodes in wheat. On the other hand, the reduction in the expression levels of ABA biosynthetic genes and ABA content in stem nodes during waterlogging supports that ABA acts as a negative regulator of nodal root formation in wheat (Nguyen et al., 2018).

Maize is sensitive to waterlogging stress and the induction of CRs is an adaptive response in some genotypes (Zhai et al., 2013). Genes belonging to group VII of the ethylene response factor (ERF) family control flooding responses in several species, such as rice (Gibbs et al., 2015). The maize genome contains 19 genes encoding ERF-VIIs and a significant association was found between the allelic variation of one of them, *ZmEREB180*, and the survival rate under long-term waterlogging stress at the seedling stage in a highly diverse maize germplasm collection (Yu et al., 2019). Indeed, the nucleotide variation in its 5′-UTR resulted in altered *ZmEREB180* expression among different maize genotypes, which was correlated with their waterlogging-tolerance, and the *ZmEREB180* overexpression enhanced CR initiation under waterlogged conditions (Yu et al., 2019). Ethylene is a known signal mediating flooding responses and it has a relevant role in AR induction (Steffens and Rasmussen, 2016). ERF-VIIs are ethylene-inducible and have been shown to function as homeostatic sensors of hypoxia (Licausi et al., 2011). A recent report demonstrated that stem-cell activity in the shoot apical meristem niche requires low-oxygen levels to sustain leaf organogenesis (Weits et al., 2019). In addition, ROS homeostasis has been identified as a factor regulating the transition between proliferating and arrested cells during LR development in Arabidopsis (Fernández-Marcos et al., 2017). These interesting results suggest that ROS might act as an endogenous diffusible molecule with important signaling functions acting on a dose-dependent manner. An intriguing possibility is that low-oxygen conditions in the submerged stems might directly regulate the observed reactivation of dormant AR primordia, a hypothesis that now could be experimentally addressed.

In other species, such as tomato (Vidoz et al., 2010) or cucumber (Xu X. et al., 2016), ARs developed *de novo* as a result of waterlogging stress. In cucumber (*Cucumis sativus*), the waterlogging tolerant landrace, “Zaoer-N,” adapted to waterlogging stress by developing a large number of ARs in the hypocotyl, while almost no ARs were generated in the waterlogging-sensitive cultivar “Pepino” (Xu X. et al., 2016). Extensive transcriptome and proteome reprogramming was observed in these two contrasting cucumber genotypes during waterlogging, which revealed candidate factors that might contribute to the AR primordia initiation in the tolerant line (Xu X. et al., 2016; Xu et al., 2017a). To gain additional insight into the genetic basis of this response, QTL mapping was performed in six generations derived from crosses between the two contrasting lines “Zaoer-N” and “Pepino” (Xu et al., 2017b). A major QTL involved in AR number variation, *ARN6.1*, was narrowed down to a small genomic interval (Xu et al., 2017b). Further fine mapping of the *ARN6.1* locus through bulked segregant analysis coupled to whole genome sequencing (BSA-Seq) and the study of haplotype diversity in a wide collection of cucumber inbred lines identified a causal polymorphism in the coding region of *CsARN6.1* responsible for the dominant waterlogging tolerance of “Zaoer-N” (Xu et al., 2018). The AAA-type ATPase family protein of the “Pepino” cultivar encoded by this gene (CsARN6.1^Gly^) was not functional, and transgenic cucumber lines expressing the CsARN6.1^Asp^ allele from “Zaoer-N” exhibited a significant increase in AR numbers under waterlogging conditions compared with that of the waterlogging-sensitive lines (Xu et al., 2018). An indirect link was proposed between CsARN6.1^Asp^ and H_2_O_2_ accumulation, which is a known trigger of AR formation in several species (Li et al., 2017). In a subsequent study, the authors showed that waterlogging induced ROS accumulation in the cambium of tolerant “Zaoer-N” hypocotyls, while inhibiting H_2_O_2_ production suppressed AR formation in waterlogging conditions (Qi et al., 2019). In addition, waterlogging-induced AR formation was stimulated by ethylene through the early upregulation of ethylene biosynthesis genes (Qi et al., 2019). In their model, ethylene stimulates the transport and accumulation of auxin in the submerged hypocotyl; and that both ethylene and auxin generate ROS, which directly or indirectly leads to AR formation (Qi et al., 2019).

## Novel Insights Into microRNA Regulation of AR Formation

In Arabidopsis, light-stress induced AR formation in the hypocotyl is regulated by a subtle balance of three activator and repressor *ARF* genes (Gutierrez et al., 2009). ARF17, a target of microRNA160 (miR160), is a negative regulator of AR formation, while ARF6 and ARF8, targets of miR167, are positive regulators of adventitious rooting, which function in a complex regulatory loop at both transcriptional and posttranscriptional level (Gutierrez et al., 2009). In the presence of high auxin levels, two F-box proteins from the TRANSPORT INHIBITOR1/AUXIN-SIGNALING F-BOX PROTEIN (TIR1/AFB) family, TIR1 and AFB2, promote 26S proteasome-mediated degradation of the auxin/indole-3-acetic acid (Aux/IAA) corepressors IAA6, IAA9 and IAA17, which in turn allows the transcriptional activity of ARF6 and ARF8 in the presence of auxin (Lakehal et al., 2019). As a result, ARF6 and ARF8 upregulate *GRETCHEN HAGEN3.3* (*GH3.3*), *GH3.5*, and *GH3.6* expression, which encode the acyl-acid-amido synthetases involved in the negative regulation of active JA levels that inhibit AR formation in the hypocotyl (Gutierrez et al., 2012; Figure 2C).

An integrated analysis of miRNAs and their target gene expression has been recently performed in poplar softwood cuttings to identify the functional miRNA-target modules involved in AR development at the genome-wide level (Cai et al., 2019). Among the miRNA-target pairs identified, poplar miR167a and its targets *PeARF6s* and *PeARF8s* were subjected to functional validation. miR167a negatively regulates AR formation in poplar, whereas overexpression of a miR167-resistant form of *PeARF8.1* promotes AR formation, suggesting that the regulatory mechanism of miR167-ARF8 during AR formation may be conserved (Cai et al., 2019). On the other hand, *PeARF17.1* and *PeARF17.2* are targeted by miR160a in poplar, and the overexpression of their miR160-resistant versions promote wound-induced AR formation (Liu et al., 2020), which indicates a highly complex regulatory mechanism of AR formation in this species. In lotus (*Nelumbo nucifera*) seedlings, ARs usually emerge on the hypocotyls or at the internodes of the rhizome underground. Expression profile of miRNAs during different stages of AR formation in this species identified 240 upregulated and 70 downregulated miRNAs at the induction stage. Several of these miRNAs were related to plant hormone metabolism, particularly auxin and brassinosteroids (BRs; Libao et al., 2019). Three miRNAs (miR157a-5p, miR9748, and miR2105) were involved in regulating the BR receptor gene *BRASSINOSTEROID INSENSITIVE1*, and four miRNAs regulated auxin transduction elements, such as TIR1 (miR393b-5p) and ARFs (miR160a and miR9748), as well as auxin target genes, such as GH3 (miR171d-5p) and SAUR (miR162-3p) (Libao et al., 2019).

The increase in AR formation and the decrease in shoot growth are a general response of plants to a mineral deficiency. Several miRNAs were differentially expressed under mineral deficiency (N, P, K, and S) conditions in rice root and shoot tissues (Grewal et al., 2018). Based on previous experimental evidence, the regulatory interactions that connect molecular events to phenotypic changes were anticipated. Some members of OsmiR167 were downregulated in the shoot but were upregulated in the root under phosphate deficiency. OsmiR167 targets and degrades *OsARF8* that, in turn, lowers OsGH3.2 function and increases the active auxin levels in the root, which might indirectly enhance CR formation (Grewal et al., 2018). On the other hand, sulfur deficiency induced OsmiR394 expression, which targets and degrades the F-box protein OsFBX32 that in turn stabilizes the Aux/IAA auxin repressors OsIAA3 and OsAXR3, leading to CR growth inhibition (Grewal et al., 2018).

In Arabidopsis, miR156 acts by repressing the expression of 11 SQUAMOSA PROMOTER BINDING PROTEIN-LIKE (SPL) genes (Xing et al., 2013). A role for miR156 in AR formation has been proposed based on the reduced number of wound-induced ARs of plants transformed with *35S::MIM156*, which blocks the activity of miR156 and causes an increase in SPL expression, providing a plausible explanation for the observation that rooting capacity declines along with plant age (Xu M. et al., 2016; Massoumi et al., 2017). Recent data indicated a more complex scenario for SPL regulation during wound-induced AR formation (Ye et al., 2020). Here, the authors used the Arabidopsis leaf explant experimental system to provide a mechanistic model for *de novo* AR formation where the wound signal activates a subset of AP2/ERF transcription factors that induce auxin biosynthesis, such as ABR1, ERF109, ERF115, and RAP2.6L, among others. In older leaves, SPL2, 10, and 11 directly bind to the promoters of these AP2/ERFs and attenuate their induction, thereby dampening auxin accumulation at the wound (Ye et al., 2020; Figure 2D).

Recently, additional evidence for the key role of miRNAs in regulating AR formation came from the molecular identification of the *crown root defect1* (*crd1*) mutant in rice (Zhu et al., 2019). *CRD1* encodes the functional ortholog of Arabidopsis HASTY (HST), known to be important in miRNA biogenesis, function and transport (Mee et al., 2005). The altered crown root development of *crd1* was phenocopied by target mimicry of miR156, which upregulates several genes of the SQUAMOSA PROMOTER BINDING PROTEIN-LIKE (SPL) transcription factor family (Zhu et al., 2019). Based on the complementary root system architecture of *hst* and *crd1* mutants, it is tempting to speculate that the miR156-SPL module play important roles in the development of the dicot taproot vs. the monocot fibrous root systems, which deserve further study.

## Concluding Remarks

Based on the fossil record and phylogenetic evidence, we propose AR formation as the default state of root development in plants. Recent molecular approaches using different species led to the identification of some of the conserved gene regulatory networks involved in the early steps of AR formation. The basic module for AR initiation consists of a hierarchy of transcription factors belonging to the ARF and WOX families, which act downstream of local auxin gradients and, in turn, regulate the activity of LBD and AP2/ERF transcription factors. Downstream regulation results in altering endogenous hormone homeostasis, particularly that of auxin and cytokinins, which reinforce signaling toward the reactivation of resident root stem cells, likely through controlling the local concentration of ROS. The environmental information is then integrated into these developmental modules through additional hormonal regulation (ethylene, ABA, and JA) and miRNA regulation. Despite all the recent advances in this field, little is known about the molecular mechanisms controlling the early specification of resident stem cells within adult tissues and the signals that maintain them in a quiescent state until their reprogramming is required for AR initiation.

## Author Contributions

JMP-P was responsible for conceptualization and supervision, was involved in the review and editing of the manuscript, and provided the funding acquisition. MM and JMP-P performed the formal analysis and wrote the original draft. Both authors contributed to the article and approved the submitted version.

## Conflict of Interest

The authors declare that the research was conducted in the absence of any commercial or financial relationships that could be construed as a potential conflict of interest.

## References

[B1] Atkinson J. A. Rasmussen A. Traini R. Voß U. Sturrock C. Mooney S. J. (2014). Branching out in roots: uncovering form, function, and regulation. *Plant Physiol.* 166 538–550. 10.1104/pp.114.245423 25136060 PMC4213086

[B2] Augstein F. Carlsbecker A. (2018). Getting to the roots: a developmental genetic view of root anatomy and function from Arabidopsis to lycophytes. *Front. Plant Sci.* 9:1410. 10.3389/fpls.2018.01410 30319672 PMC6167918

[B3] Bellini C. Pacurar D. I. Perrone I. (2014). Adventitious roots and lateral roots: similarities and differences. *Annu. Rev. Plant Biol.* 65 639–666. 10.1146/annurev-arplant-050213-035645 24555710

[B4] Burton A. L. Johnson J. M. Foerster J. M. Hirsch C. N. Buell C. R. Hanlon M. T. (2014). QTL mapping and phenotypic variation for root architectural traits in maize (*Zea mays* L.). *Theor. Appl. Genet.* 127 2293–2311. 10.1007/s00122-014-2353-4 25230896

[B5] Bustillo-Avendaño E. Ibáñez S. Sanz O. Barros J. A. S. Gude I. Perianez-Rodriguez J. (2018). Regulation of hormonal control, cell reprogramming and patterning during de novo root organogenesis. *Plant Physiol.* 176 1709–1727. 10.1104/pp.17.00980 29233938 PMC5813533

[B6] Cai H. Yang C. Liu S. Qi H. Wu L. Xu L.-A. (2019). MiRNA-target pairs regulate adventitious rooting in *Populus*: a functional role for miR167a and its target Auxin response factor 8. *Tree Physiol.* 39 1922–1936. 10.1093/treephys/tpz085 31504994

[B7] Dawood T. Rieu I. Wolters-Arts M. Derksen E. B. Mariani C. Visser E. J. W. (2014). Rapid flooding-induced adventitious root development from preformed primordia in *Solanum dulcamara*. *AoB Plants* 6:plt058. 10.1093/aobpla/plt058 24790121 PMC3922303

[B8] Dawood T. Yang X. Visser E. J. W. Te Beek T. A. H. Kensche P. R. Cristescu S. M. (2016). A co-opted hormonal cascade activates dormant adventitious root primordia upon flooding in *Solanum dulcamara*. *Plant Physiol.* 170 2351–2364. 10.1104/pp.15.00773 26850278 PMC4825138

[B9] Dolzblasz A. Nardmann J. Clerici E. Causier B. van der Graaff E. Chen J. (2016). Stem cell regulation by *Arabidopsis WOX* genes. *Mol. Plant* 9 1028–1039. 10.1016/j.molp.2016.04.007 27109605

[B10] Druege U. Hilo A. Pérez-Pérez J. M. Klopotek Y. Acosta M. Shahinnia F. (2019). Molecular and physiological control of adventitious rooting in cuttings: phytohormone action meets resource allocation. *Ann. Bot.* 123 929–949. 10.1093/aob/mcy234 30759178 PMC6589513

[B11] Dubrovsky J. G. Gambetta G. A. Hernández-Barrera A. Shishkova S. González I. (2006). Lateral root initiation in *Arabidopsis*: developmental window, spatial patterning, density and predictability. *Ann. Bot.* 97 903–915. 10.1093/aob/mcj604 16390845 PMC2803408

[B12] Fernández-Marcos M. Desvoyes B. Manzano C. Liberman L. M. Benfey P. N. del Pozo J. C. (2017). Control of Arabidopsis lateral root primordium boundaries by MYB36. *New Phytol.* 213 105–112. 10.1111/nph.14304 27891649 PMC5126979

[B13] Fujinami R. Yamada T. Nakajima A. Takagi S. Idogawa A. Kawakami E. (2017). Root apical meristem diversity in extant lycophytes and implications for root origins. *New Phytol.* 215 1210–1220. 10.1111/nph.14630 28585243

[B14] Gao Y. Lynch J. P. (2016). Reduced crown root number improves water acquisition under water deficit stress in maize (*Zea mays* L.). *J. Exp. Bot.* 67 4545–4557. 10.1093/jxb/erw243 27401910 PMC4973737

[B15] Gibbs D. J. Conde J. V. Berckhan S. Prasad G. Mendiondo G. M. Holdsworth M. J. (2015). Group VII ethylene response factors coordinate oxygen and nitric oxide signal transduction and stress responses in plants. *Plant Physiol.* 169 23–31. 10.1104/pp.15.00338 25944828 PMC4577381

[B16] Gonin M. Bergougnoux V. Nguyen T. D. Gantet P. Champion A. (2019). What makes adventitious roots? *Plants* 8:240. 10.3390/plants8070240 31336687 PMC6681363

[B17] Grewal R. K. Saraf S. Deb A. Kundu S. (2018). Differentially expressed microRNAs link cellular physiology to phenotypic changes in rice under stress conditions. *Plant Cell Physiol.* 59 2143–2154. 10.1093/pcp/pcy136 30010993

[B18] Gutierrez L. Bussell J. D. Pacurar D. I. Schwambach J. Pacurar M. Bellini C. (2009). Phenotypic plasticity of adventitious rooting in *Arabidopsis* is controlled by complex regulation of AUXIN RESPONSE FACTOR transcripts and microRNA abundance. *Plant Cell* 21 3119–3132. 10.1105/tpc.108.064758 19820192 PMC2782293

[B19] Gutierrez L. Mongelard G. Floková K. Pacurar D. I. Novák O. Staswick P. (2012). Auxin controls *Arabidopsis* adventitious root initiation by regulating jasmonic acid homeostasis. *Plant Cell* 24 2515–2527. 10.1105/tpc.112.099119 22730403 PMC3406919

[B20] Gutjahr C. Sawers R. J. H. Marti G. Andrés-Hernández L. Yang S. Y. Casieri L. (2015). Transcriptome diversity among rice root types during asymbiosis and interaction with arbuscular mycorrhizal fungi. *Proc. Natl. Acad. Sci. U.S.A.* 112 6754–6759. 10.1073/pnas.1504142112 25947154 PMC4450400

[B21] Hetherington A. J. Dolan L. (2018). Stepwise and independent origins of roots among land plants. *Nature* 561 235–238. 10.1038/s41586-018-0445-z 30135586 PMC6175059

[B22] Hochholdinger F. (2016). Untapping root system architecture for crop improvement. *J. Exp. Bot.* 67 4431–4433. 10.1093/jxb/erw262 27493225 PMC4973748

[B23] Hochholdinger F. Yu P. Marcon C. (2018). Genetic control of root system development in maize. *Trends Plant Sci.* 23 79–88. 10.1016/j.tplants.2017.10.004 29170008

[B24] Hu X. Xu L. (2016). Transcription factors WOX11/12 directly activate WOX5/7 to promote root primordia initiation and organogenesis. *Plant Physiol.* 172 2363–2373. 10.1104/pp.16.01067 27784768 PMC5129711

[B25] Inukai Y. Sakamoto T. Ueguchi-Tanaka M. Shibata Y. Gomi K. Umemura I. (2005). crown rootless1, which is essential for crown root formation in rice, is a target of an AUXIN RESPONSE FACTOR in Auxin signaling. *Plant Cell* 17 1387–1396. 10.1105/tpc.105.030981 15829602 PMC1091762

[B26] Jing T. Ardiansyah R. Xu Q. Xing Q. Müller-Xing R. (2020). Reprogramming of cell fate during root regeneration by transcriptional and epigenetic networks. *Front. Plant Sci.* 11:317. 10.3389/fpls.2020.00317 32269581 PMC7112134

[B27] Kenrick P. Strullu-Derrien C. (2014). The origin and early evolution of roots. *Plant Physiol.* 166 570–580. 10.1104/PP.114.244517 25187527 PMC4213089

[B28] Kitomi Y. Ito H. Hobo T. Aya K. Kitano H. Inukai Y. (2011). The auxin responsive AP2/ERF transcription factor CROWN ROOTLESS5 is involved in crown root initiation in rice through the induction of OsRR1, a type-A response regulator of cytokinin signaling. *Plant J.* 67 472–484. 10.1111/j.1365-313X.2011.04610.x 21481033

[B29] Kloth K. J. Wiegers G. L. Busscher-Lange J. van Haarst J. C. Kruijer W. Bouwmeester H. J. (2016). AtWRKY22 promotes susceptibility to aphids and modulates salicylic acid and jasmonic acid signalling. *J. Exp. Bot.* 67 3383–3396. 10.1093/jxb/erw159 27107291 PMC4892728

[B30] Kumar S. Stecher G. Li M. Knyaz C. Tamura K. (2018). MEGA X: molecular evolutionary genetics analysis across computing platforms. *Mol. Biol. Evol.* 35 1547–1549. 10.1093/molbev/msy096 29722887 PMC5967553

[B31] Lakehal A. Bellini C. (2019). Control of adventitious root formation: insights into synergistic and antagonistic hormonal interactions. *Physiol. Plant.* 165 90–100. 10.1111/ppl.12823 30159890

[B32] Lakehal A. Chaabouni S. Cavel E. Le Hir R. Ranjan A. Raneshan Z. (2019). A molecular framework for the control of adventitious rooting by the TIR1/AFB2-Aux/IAA-dependent auxin signaling in *Arabidopsis*. *Mol. Plant* 12 1499–1514. 10.1016/J.MOLP.2019.09.001 31520787

[B33] Li P. Zhang Y. Yin S. Zhu P. Pan T. Xu Y. (2018). QTL-by-environment interaction in the response of maize root and shoot traits to different water regimes. *Front. Plant Sci.* 9:229. 10.3389/fpls.2018.00229 29527220 PMC5829059

[B34] Li S.-W. Leng Y. Shi R.-F. (2017). Transcriptomic profiling provides molecular insights into hydrogen peroxide-induced adventitious rooting in mung bean seedlings. *BMC Genomics* 18:188. 10.1186/s12864-017-3576-y 28212614 PMC5316208

[B35] Libao C. Huiying L. Yuyan H. Shuyan L. (2019). Transcriptome analysis of miRNAs expression reveals novel insights into adventitious root formation in lotus (*Nelumbo nucifera* Gaertn.). *Mol. Biol. Rep.* 46 2893–2905. 10.1007/s11033-019-04749-z 30864113

[B36] Licausi F. Kosmacz M. Weits D. A. Giuntoli B. Giorgi F. M. Voesenek L. A. C. J. (2011). Oxygen sensing in plants is mediated by an N-end rule pathway for protein destabilization. *Nature* 479 419–422. 10.1038/nature10536 22020282

[B37] Liu H. J. Wang S. F. Yu X. B. Yu J. He X. W. Zhang S. L. (2005). ARL1, a LOB-domain protein required for adventitious root formation in rice. *Plant J.* 43 47–56. 10.1111/j.1365-313X.2005.02434.x 15960615

[B38] Liu J. Sheng L. Xu Y. Li J. Yang Z. Huang H. (2014). *WOX11* and *12* are involved in the first-step cell fate transition during de novo root organogenesis in *Arabidopsis*. *Plant Cell* 26 1081–1093. 10.1105/tpc.114.122887 24642937 PMC4001370

[B39] Liu S. Wang J. Wang L. Wang X. Xue Y. Wu P. (2009). Adventitious root formation in rice requires OsGNOM1 and is mediated by the OsPINs family. *Cell Res.* 19 1110–1119. 10.1038/cr.2009.70 19546891

[B40] Liu S. Yang C. Wu L. Cai H. Li H. Xu M. (2020). The *peu-miR160a-PeARF17.1/PeARF17.2* module participates in the adventitious root development of poplar. *Plant Biotechnol. J.* 18 457–469. 10.1111/pbi.13211 31314168 PMC6953198

[B41] Liu W. Xu L. (2018). Recruitment of IC-*WOX* genes in root evolution. *Trends Plant Sci.* 23 490–496. 10.1016/J.TPLANTS.2018.03.011 29680635

[B42] Martínez-de la Cruz E. García-Ramírez E. Vázquez-Ramos J. M. Reyes de la Cruz H. López-Bucio J. (2015). Auxins differentially regulate root system architecture and cell cycle protein levels in maize seedlings. *J. Plant Physiol.* 176 147–156. 10.1016/j.jplph.2014.11.012 25615607

[B43] Massoumi M. Krens F. A. Visser R. G. F. De Klerk G.-J. M. (2017). Azacytidine and miR156 promote rooting in adult but not in juvenile *Arabidopsis* tissues. *J. Plant Physiol.* 208 52–60. 10.1016/J.JPLPH.2016.10.010 27889521

[B44] Mee Y. P. Wu G. Gonzalez-Sulser A. Vaucheret H. Poethig R. S. (2005). Nuclear processing and export of microRNAs in *Arabidopsis*. *Proc. Natl. Acad. Sci. U.S.A.* 102 3691–3696. 10.1073/pnas.0405570102 15738428 PMC553294

[B45] Nguyen T.-N. Tuan P. A. Mukherjee S. Son S. Ayele B. T. (2018). Hormonal regulation in adventitious roots and during their emergence under waterlogged conditions in wheat. *J. Exp. Bot.* 69 4065–4082. 10.1093/jxb/ery190 29788353 PMC6054230

[B46] Péret B. De Rybel B. Casimiro I. Benková E. Swarup R. Laplaze L. (2009). *Arabidopsis* lateral root development: an emerging story. *Trends Plant Sci.* 14 399–408. 10.1016/J.TPLANTS.2009.05.002 19559642

[B47] Pires N. D. Dolan L. (2012). Morphological evolution in land plants: new designs with old genes. *Philos. Trans. R. Soc. B Biol. Sci.* 367 508–518. 10.1098/rstb.2011.0252 22232763 PMC3248709

[B48] Qi X. Li Q. Ma X. Qian C. Wang H. Ren N. (2019). Waterlogging-induced adventitious root formation in cucumber is regulated by ethylene and auxin through reactive oxygen species signalling. *Plant Cell Environ.* 42 1458–1470. 10.1111/pce.13504 30556134

[B49] Sebastian J. Yee M. C. Viana W. G. Rellán-Álvarez R. Feldman M. Priest H. D. (2016). Grasses suppress shoot-borne roots to conserve water during drought. *Proc. Natl. Acad. Sci. U.S.A.* 113 8861–8866. 10.1073/pnas.1604021113 27422554 PMC4978293

[B50] Skubacz A. Daszkowska-Golec A. Szarejko I. (2016). The role and regulation of ABI5 (ABA-insensitive 5) in plant development, abiotic stress responses and phytohormone crosstalk. *Front. Plant Sci.* 7:1884. 10.3389/fpls.2016.01884 28018412 PMC5159420

[B51] Steffens B. Kovalev A. Gorb S. N. Sauter M. (2012). Emerging roots alter epidermal cell fate through mechanical and reactive oxygen species signaling. *Plant Cell* 24 3296–3306. 10.1105/tpc.112.101790 22904148 PMC3462632

[B52] Steffens B. Rasmussen A. (2016). The physiology of adventitious roots. *Plant Physiol.* 170 603–617. 10.1104/pp.15.01360 26697895 PMC4734560

[B53] Steffens B. Wang J. Sauter M. (2006). Interactions between ethylene, gibberellin and abscisic acid regulate emergence and growth rate of adventitious roots in deepwater rice. *Planta* 223 604–612. 10.1007/s00425-005-0111-1 16160845

[B54] Sun B. Gao Y. Lynch J. P. (2018). Large crown root number improves topsoil foraging and phosphorus acquisition. *Plant Physiol.* 177 90–104. 10.1104/pp.18.00234 29618638 PMC5933112

[B55] Sun P. Jia H. Zhang Y. Li J. Lu M. Hu J. (2019). Deciphering genetic architecture of adventitious root and related shoot traits in *Populus* using QTL mapping and RNA-seq data. *Int. J. Mol. Sci.* 20:6114. 10.3390/ijms20246114 31817197 PMC6941115

[B56] Tai H. Lu X. Opitz N. Marcon C. Paschold A. Lithio A. (2016). Transcriptomic and anatomical complexity of primary, seminal, and crown roots highlight root type-specific functional diversity in maize (*Zea mays* L.). *J. Exp. Bot.* 67 1123–1135. 10.1093/jxb/erv513 26628518 PMC4753849

[B57] Takehisa H. Sato Y. Igarashi M. Abiko T. Antonio B. A. Kamatsuki K. (2012). Genome-wide transcriptome dissection of the rice root system: implications for developmental and physiological functions. *Plant J.* 69 126–140. 10.1111/j.1365-313X.2011.04777.x 21895812

[B58] Taramino G. Sauer M. Stauffer J. L. Multani D. Niu X. Sakai H. (2007). The maize (*Zea mays* L.) RTCS gene encodes a LOB domain protein that is a key regulator of embryonic seminal and post-embryonic shoot-borne root initiation. *Plant J.* 50 649–659. 10.1111/j.1365-313X.2007.03075.x 17425722

[B59] Vidoz M. L. Loreti E. Mensuali A. Alpi A. Perata P. (2010). Hormonal interplay during adventitious root formation in flooded tomato plants. *Plant J.* 63 551–562. 10.1111/j.1365-313X.2010.04262.x 20497380

[B60] Wang M. Le Moigne M. A. Bertheloot J. Crespel L. Perez-Garcia M. D. Ogé L. (2019). BRANCHED1: a key hub of shoot branching. *Front. Plant Sci.* 10:76. 10.3389/fpls.2019.00076 30809235 PMC6379311

[B61] Wang P. Ma L. Wang S. Li L. Wang Q. Yang R. (2019). Identification and analysis of a candidate *WRKY* transcription factor gene affecting adventitious root formation using association mapping in *Catalpa* Scop. *DNA Cell Biol.* 38 297–306. 10.1089/dna.2018.4528 30676076

[B62] Weits D. A. Kunkowska A. B. Kamps N. C. W. Portz K. M. S. Packbier N. K. Nemec Venza Z. (2019). An apical hypoxic niche sets the pace of shoot meristem activity. *Nature* 569 714–717. 10.1038/s41586-019-1203-6 31092919

[B63] Xing S. Salinas M. Garcia-Molina A. Höhmann S. Berndtgen R. Huijser P. (2013). *SPL8* and miR156-targeted *SPL* genes redundantly regulate Arabidopsis gynoecium differential patterning. *Plant J.* 75 566–577. 10.1111/tpj.12221 23621152

[B64] Xu C. Tai H. Saleem M. Ludwig Y. Majer C. Berendzen K. W. (2015). Cooperative action of the paralogous maize lateral organ boundaries (LOB) domain proteins RTCS and RTCL in shoot-borne root formation. *New Phytol.* 207 1123–1133. 10.1111/nph.13420 25902765

[B65] Xu M. Hu T. Zhao J. Park M.-Y. Y. Earley K. W. Wu G. (2016). Developmental functions of miR156-regulated *SQUAMOSA PROMOTER BINDING PROTEIN-LIKE (SPL)* genes in *Arabidopsis thaliana*. *PLoS Genet.* 12:e1006263. 10.1371/journal.pgen.1006263 27541584 PMC4991793

[B66] Xu X. Ji J. Ma X. Xu Q. Qi X. Chen X. (2016). Comparative proteomic analysis provides insight into the key proteins involved in cucumber (*Cucumis sativus* L.) adventitious root emergence under waterlogging stress. *Front. Plant Sci.* 7:1515. 10.3389/fpls.2016.01515 27790230 PMC5062059

[B67] Xu X. Chen M. Ji J. Xu Q. Qi X. Chen X. (2017a). Comparative RNA-seq based transcriptome profiling of waterlogging response in cucumber hypocotyls reveals novel insights into the de novo adventitious root primordia initiation. *BMC Plant Biol.* 17:129. 10.1186/s12870-017-1081-8 28747176 PMC5530484

[B68] Xu X. Ji J. Xu Q. Qi X. Chen X. (2017b). Inheritance and quantitative trail loci mapping of adventitious root numbers in cucumber seedlings under waterlogging conditions. *Mol. Genet. Genomics* 292 353–364. 10.1007/s00438-016-1280-2 27988808

[B69] Xu X. Ji J. Xu Q. Qi X. Weng Y. Chen X. (2018). The major-effect quantitative trait locus *CsARN6.1* encodes an AAA ATPase domain-containing protein that is associated with waterlogging stress tolerance by promoting adventitious root formation. *Plant J.* 93 917–930. 10.1111/tpj.13819 29315927

[B70] Yamauchi T. Watanabe K. Fukazawa A. Mori H. Abe F. Kawaguchi K. (2014). Ethylene and reactive oxygen species are involved in root aerenchyma formation and adaptation of wheat seedlings to oxygen-deficient conditions. *J. Exp. Bot.* 65 261–273. 10.1093/jxb/ert371 24253196 PMC3883296

[B71] Yang X. Jansen M. J. Zhang Q. Sergeeva L. Ligterink W. Mariani C. (2018). A disturbed auxin signaling affects adventitious root outgrowth in *Solanum dulcamara* under complete submergence. *J. Plant Physiol.* 224–225 11–18. 10.1016/j.jplph.2018.03.006 29574325

[B72] Ye B. B. Shang G. D. Pan Y. Xu Z. G. Zhou C. M. Mao Y. B. (2020). AP2/ERF transcription factors integrate age and wound signals for root regeneration. *Plant Cell* 32 226–241. 10.1105/tpc.19.00378 31649122 PMC6961627

[B73] Yu F. Liang K. Fang T. Zhao H. Han X. Cai M. (2019). A group VII ethylene response factor gene, *ZmEREB180*, coordinates waterlogging tolerance in maize seedlings. *Plant Biotechnol. J.* 17 2286–2298. 10.1111/pbi.13140 31033158 PMC6835127

[B74] Yu J. Zhang Y. Liu W. Wang H. Wen S. Zhang Y. (2020). Molecular evolution of auxin-mediated root initiation in plants. *Mol. Biol. Evol.* 37 1387–1393. 10.1093/molbev/msz202 31504735

[B75] Yu P. Baldauf J. A. Lithio A. Marcon C. Nettleton D. Li C. (2016). Root type-specific reprogramming of maize pericycle transcriptomes by local high nitrate results in disparate lateral root branching patterns. *Plant Physiol.* 170 1783–1798. 10.1104/pp.15.01885 26811190 PMC4775145

[B76] Zhai L. Liu Z. Zou X. Jiang Y. Qiu F. Zheng Y. (2013). Genome-wide identification and analysis of microRNA responding to long-term waterlogging in crown roots of maize seedlings. *Physiol. Plant.* 147 181–193. 10.1111/j.1399-3054.2012.01653.x 22607471

[B77] Zhang M. Kong X. Xu X. Li C. Tian H. Ding Z. (2015). Comparative transcriptome profiling of the maize primary, crown and seminal root in response to salinity stress. *PLoS One* 10:e0121222. 10.1371/journal.pone.0121222 25803026 PMC4372355

[B78] Zhao Y. Cheng S. Song Y. Huang Y. Zhou S. Liu X. (2015). The interaction between rice ERF3 and WOX11 promotes crown root development by regulating gene expression involved in cytokinin signaling. *Plant Cell* 27 2469–2483. 10.1105/tpc.15.00227 26307379 PMC4815106

[B79] Zhao Y. Hu Y. Dai M. Huang L. Zhou D. X. (2009). The WUSCHEL-related homeobox gene *WOX11* is required to activate shoot-borne crown root development in rice. *Plant Cell* 21 736–748. 10.1105/tpc.108.061655 19258439 PMC2671696

[B80] Zhu J. Li Y. Lin J. Wu Y. Guo H. Shao Y. (2019). CRD1, an Xpo1 domain protein, regulates miRNA accumulation and crown root development in rice. *Plant J.* 100 328–342. 10.1111/tpj.14445 31257621

