# Supplementary material for: Understanding of Adventitious Root Formation: What Can We Learn From Comparative Genetics?
Source: Front Plant Sci. 2020 Oct 6;11:582020. doi: 10.3389/fpls.2020.582020 (PMC7573222; doi:10.3389/fpls.2020.582020)
Supplement: Supplementary file 1 [file Table_1.DOCX]

*>Marchantia_polymorpha*

*atgtcaccacaaacggagactaaagcaggtgttggattcaaagctggtgttaaagattatcgattaaattattacactccggattatgagaccaaggatacggatattttagcagcatttagaatgactcctcaacctggagttccagcagaagaagcaggagcagcagttgctgctgagtcttcaactggtacatggactacagtttggactgatggtcttactaaccttgatcgttataaaggtcgatgctatgatattgaccctgttcctggagaagataatcaatatattgcttatgtagcttatcctttagatttatttgaagaaggttctgttacaaatatgtttacttcaattgtaggtaatgtatttgggtttaaagctttaagagcgttacgtcttgaagatttaagaattcctccagcttacacaaaaactttccaaggtcctcctcatggtattcaagttgagagagataaattaaacaaatatggtcgtcctttattaggatgtactattaaaccaaaattaggtttatctgctaaaaactatggtagagctgtatatgaatgtcttcgtggtggacttgattttactaaagatgatgaaaacgtaaattctcaaccatttatgcgttggagagatcgtttcttatttgtagcagaagctatttttaaatctcaagcagaaactggagaaatcaaaggacattatttaaatgctactgcaggtacatgtgaagaaatgctaaaaagagcagcatgtgctagagagttaggtgtaccaattgttatgcacgactacttaactggtggtttcactgcaaatactactctagctttttattgccgtgacaatggtttacttcttcatattcaccgtgcaatgcatgcagttattgatagacaaaaaaatcatggtatccacttccgtgtattagcaaaagctttacgtatgtctggtggagatcatattcacgctggtactgttgtaggtaaacttgaaggagaccgtaacgtaactttaggtttcgtagatttacttcgtgatgactatattgaaaaagatagaagtcgtggtatttatttcacacaagattgggtttctttacctggtgttttccctgtagcatctggtgggatccatgtttggcatatgcctgctttaactgaaatttttggcgatgactctgttttacaatttggtggtggaactttaggtcatccttggggtaacgcacctggtgcagttgctaaccgagtttcgttagaagcttgtgtacaagcacgtaatgaaggtcgtgaccttgctcgtgaaggaaatgaaattattcgtgaagcttgcaaatggagtcctgagttatctgctgcttgtgaagtttggaaagaaattaaatttgaatttgatattattgatactttgtaa*

*>Ceratopteris_richardii*

*atgtcgccacaaacggagactaaagcaggtgttggatttcaagctggtgtcaaagattaccgattgacttattacactcccgagtataaggtcaaagatactgatatcttagccgcttttcgaatgaccccacaacccggagtaccagctgaagaggccggagctgcggtggctgcggaatcctctacaggtacatggaccacagtatggacagacggacttactagtcttgatcgatacaaaggtcggtgctacgatattgaacccgttgccggggaggaaaaccagtatattgcatatgtagcgtaccccttggatttatttgaggaagggtccgtcaccaatatgctgacttctattgtaggtaatgtttttggattcaaggccttacgcgctctgcgcctggaagacctacggattcctcctgcttattccaagacttttcttgggcctcctcacggtattcaggtcgaaagagataaattaaacaaatatggtcgtcctctattgggatgtacaatcaagccaaaattgggcttgtctgctaaaaattatggtagagcagtttacgaatgccttcgtggtggacttgatttcacaaaagatgatgaaaacgttaattcccaaccgtttatgcgttggagagatcgcttttgcttcgtagccgaagctctttataaagcccagaatgaaaccggtgaaattaagggacattatttaaatgccaccgcgggcacaactgaagaaatgcttagaagagctgactatgccagtgaattgggtgcaccaatcatcatgcatgactatctgactggtggttttaccgcaaatactagtttagctatctattctagaaacaaagggctacttcttcatattcaccgagccatgcatgctgttattgatagacaaagaaatcatggtatgcatttccgtgttttagccaaagccttacgtatgtctggtggagatcatatccatgcaggaactgtggtgggcaaattagaaggtgaacgagaagttaccctgggatttgtcgatttacttcgcgacgactacattgaaaaagatcgtaagcgtggtatatacttcacccaagattgggtatctatgcctggtgtaattcccgtggcttcagggggtattcacgtatggcatatgcccgccctaaccgaaatttttggggacgattctgtcttacaatttggcgggggaacattgggacatccttggggaaatgcccctggtgccgttgctaaccgcgtagcattggaggcttgtgtgcaggctcgtaatgagggacgtgatctggctcgtgaaggtaatgagataattcgtgaagctgctcagtggagtccagaattggctgccgcatgcgaaatatggaaagcaatcaaatttgaatttgatacagttgatgtattataa*

*>Setaria_viridis*

*atgtcaccacaaacagaaactaaagcaagtgttggatttaaagctggtgttaaggattataaattgacttactacaccccggagtacgaaaccaaggatactgatatcttggcagcattccgagtaactcctcagcccggggttccgcctgaagaagcaggggctgcagtagctgcggaatcttctactggtacatggacaactgtttggactgatggacttaccagtcttgatcgttacaaaggacgatgctatcacatcgagcccgttcctggggaggcagatcaatatatctgttatatagcttatccattagacctatttgaagagggttctgttactaacatgtttacttccattgtgggtaacgtgtttggtttcaaagccctacgcgctctacgtttggaggatctacgaattcccactgcttatgcaaaaactttccaaggtccgcctcacggtatccaagttgaaagggataagttgaacaagtatggtcgtcctttattgggatgtactattaaaccaaaattgggattatccgcaaaaaattacggtagagcgtgttatgagtgtctacgcggtggacttgattttaccaaagatgatgaaaacgtaaactcacaaccatttatgcgctggagagaccgttttgtcttttgtgctgaagcaatttataaagcacaagcagaaactggtgaaattaaggggcattacttgaatgcgactgcaggtacatgcgaagaaatgattaagagagctgcatttgcaagggaattaggggttcctattgtaatgcatgactacttaactggaggattcaccgcaaatactagtttgtcttattattgccgcgacaacggcctacttcttcacattcaccgagcaatgcatgcagttattgatagacagaaaaatcatggtatgcatttccgtgtattagctaaagcattgcgtatgtcggggggagatcatatccactccggtacagtagtaggtaagttagaaggggaacgcgaaataactttaggttttgttgatttattgcgcgatgattttattgaaaaagatcgttctcgcggtatctttttcactcaggactgggcatccatgccaggtgttataccggtggcttcagggggtattcatgtttggcatatgccagctctgaccgaaatctttggagacgattctgtattacaatttggtggaggaactttaggacatccttggggaaatgcacctggtgcagcagctaatcgtgtggctttagaagcctgtgtacaagcccgtaacgaagggcgcgatcttgctcgtgaaggtaatgaaattatcaaagcagcttgcaaatggagtcctgaactagccgcagcttgtgaagtatggaaggagatcaaatttgagttcgaagcgatggataccctatag*

*>Solanum_dulcamara*

*atgtcaccacaaacagagactaaagcaagtgttggattcaaagctggtgttaaagagtacaaattgacttattatactcctgagtaccaaaccaaggatactgatatattggcagcattccgagtaactcctcaacctggagttccacctgaagaagcaggggccgcggtagctgccgaatcttctactggtacatggacaactgtatggaccgatggacttaccagtcttgatcgttacaaagggcgatgctaccgcatcgagcgtgttgttggagaaaaagatcaatatattgcttatgtagcttaccctttagacctttttgaagaaggttccgttaccaatatgttgacttccattgtaggtaatgtatttgggttcaaagccttgcgcgctctacgtctggaagatctgcgaatccctgttgcttatgttaaaactttccaaggtccgcctcatggtatccaagttgaaagagataaattgaacaagtatggtcgtcccctgttgggatgtactattaaacctaaattggggttatctgctaaaaactacggtagagctgtttatgaatgtcttcgcggtggacttgattttaccaaagatgatgagaacgtgaactcacaaccatttatgcgttggagagatcgtttcttattttgtgccgaagcactttttaaagcacagactgaaacaggtgaaatcaaagggcattacttgaatgctactgcaggtacatgcgaagaaatgatgaaaagagctgtatttgctagagaattgggcactccgatcgtaatgcatgactacttaacggggggattcaccgcaaatactaccttggctcattattgccgcgataatggtctacttcttcacatccaccgtgcaatgcatgcggttattgatagacagaagaatcatggtatgcacttccgggtattagcaaaagctttacgtatgtctggtggagatcatattcactctggtaccgtagtaggtaaacttgaaggtgaaagagacataactttgggctttgttgatttactgcgtgatgattttattgaacaagatagaagtcgcggtatttatttcactcaagattgggtttctttaccaggtgttctacctgtggcttcaggaggtattcacgtttggcatatgcctgctctgaccgagatctttggggatgattccgtactacagttcggtggaggaactttaggacatccttggggtaatgcgccaggtgccgtagctaatcgagtagctctagaagcatgtgtaaaagctcgtaatgaaggacgtgatcttgctcgggaaggtaatgagattattcgcgaggcttccaaatggagcccggaactagctgctgcttgtgaggtatggaaagagatcgtatttaattttgcagcagtggacgttttggataagtaa*

*>Solanum_lycopersicum*

*atgtcaccacaaacagagactaaagcaagtgttggattcaaagctggtgttaaagagtacaaattgacttattatactcctgagtaccaaaccaaggatactgatatattagcagcattccgagtaactcctcaacctggagttccacctgaagaagcaggggccgcggtagctgccgaatcttctactggtacatggacaactgtatggaccgatggacttaccagtcttgatcgttacaaagggcgatgctaccgcatcgagcgcgttgttggagaaaaagatcaatatattgcttatgtagcttaccctttagacctttttgaagaaggttccgttaccaatatgtttacttccattgtaggtaacgtatttgggttcaaagccctgcgcgctctacgtctggaagatctgcgaatccctcctgcttatgttaaaactttccaaggtccgcctcatgggatccaagttgaaagagataaattgaacaagtatggtcgtcccctgttgggatgtactattaaacctaaattggggttatctgcaaaaaactacggtagagctgtttatgaatgtcttcgcggtggacttgattttaccaaagatgatgagaacgtgaactcacaaccatttatgcgttggagagatcgtttcttattttgtgccgaagcactttttaaagcacagactgaaacaggtgaaatcaaagggcattacttgaatgctactgcaggtacatgcgaagaaatgatcaaaagagctgtatttgctagagaattgggcgttccgatcgtaatgcatgactacttaacggggggatttaccgcaaatactaccttggctcattattgccgagataatggtctacttcttcacatccaccgtgcaatgcatgcggttattgatagacagaagaatcatggtatccacttccgggtattagcaaaagcgttacgtatgtctggtggagatcatattcactctggtaccgtagtaggtaaacttgaaggtgaaagagacataactttgggctttgttgatttactgcgtgatgattttgttgaacaagatagaagtcgcggtatttatttcactcaagattgggtctctttaccaggtgttctacctgtggcttcaggaggtattcacgtttggcatatgcctgctctgaccgagatctttggggatgattccgtactacagttcggtggaggaactttaggacatccttggggtaatgcgccaggtgccgtagctaatcgagtagctctagaagcatgtgtaaaagctcgtaatgaaggacgtgatcttgctcgggaaggtaatgagattattcgcgaggcttgcaaatggagcccggaactagctgctgcttgtgaggtatggaaagagatcgtatttaattttgcagcagtggacgttttggataagtaa*

*>Zea_mays*

*atgtcaccacaaacagaaactaaagcaagtgttggatttaaagctggtgttaaggattataaattgacttactacaccccggagtacgaaaccaaggatactgatatcttggcagcattccgagtaactcctcagctcggggttccgcctgaagaagcaggagctgcagtagctgcggaatcttctactggtacatggacaactgtttggactgatggacttaccagtcttgatcgttacaaaggacgatgctatcacatcgagcccgttcctggggacccagatcaatatatctgttatgtagcttatccattagacctatttgaagagggttctgttactaacatgtttacttccattgtgggtaacgtatttggtttcaaagccttacgcgctctacgtttggaggatctacgaattccccctgcttattcaaaaactttccaaggtccgcctcacggtatccaagttgaaagggataagttgaacaagtacggtcgtcctttattgggatgtactattaaaccaaaattgggattatccgcaaaaaattacggtagagcgtgttatgagtgtctacgcggtggacttgattttaccaaagatgatgaaaacgtaaactcacaaccatttatgcgctggagagaccgtttcgtcttttgtgccgaagcaatttataaagcacaagccgaaactggtgaaatcaaggggcattacttgaatgcgactgcaggtacatgcgaagaaatgattaagagagctgtatttgcaagggaattaggggttcctattgtaatgcatgactacttaacaggaggattcaccgcaaatactactttgtctcattattgccgcgacaacggcctacttcttcacattcaccgagcaatgcatgcagttattgatagacagaaaaatcatggtatgcatttccgtgtattagctaaagcattgcgtatgtcggggggagatcatatccactccggtacagtagtaggtaagttagaaggggaacgcgaaataactttaggttttgttgatttattgcgcgatgattttattgaaaaagatcgttctcgcggtatctttttcactcaggactgggtatccatgccaggtgttataccggtggcttctgggggtattcatgtttggcatatgccagctctgaccgaaatctttggagatgattccgtattacaatttggtggaggaactttaggacatccttggggaaatgcacctggtgcagcagctaatcgtgtggctttagaagcctgtgtacaagctcgtaacgaagggcgcgatcttgctcgtgaaggtaatgaaattatcaaagcagcttgcaaatggagtgctgaactagccgcagcttgtgaaatatggaaggagatcaaatttgatggtttcaaagcgatggataccatataa*

*>Oryza_sativa*

*atgtcaccacaaacagaaactaaagcaagtgttggatttaaagctggtgttaaggattataaattgacttactacaccccggagtacgaaaccaaggacactgatatcttggcagcattccgagtaactcctcagccgggggttccgcccgaagaagcaggggctgcagtagctgccgaatcttctactggtacatggacaactgtttggactgatggacttaccagtcttgatcgttacaaaggccgatgctatcacatcgagcccgttgttggggaggataatcaatatatcgcttatgtagcttatccattagacctatttgaagagggttctgttactaacatgtttacttccattgtgggtaacgtatttggtttcaaagccctacgcgctctacgtctggaggatctgcgaattccccctacttattcaaaaactttccaaggtccgcctcatggtatccaagttgaaagggataagttgaacaaatacggtcgtcctttattgggatgtactattaaaccaaaattgggattatctgcaaaaaattatggtagagcatgttatgagtgtctacgcggtggacttgattttaccaaagatgatgaaaacgtaaactcacaaccatttatgcgttggagggaccgttttgtcttttgtgccgaagctatttataaatcacaggccgaaaccggtgaaattaaggggcattacttgaatgcgactgcaggtacatgcgaagaaatgattaaaagagctgtatttgcgagggaattaggggttcctattgtaatgcatgactacttaaccgggggattcaccgcaaatactagtttggctcattattgccgcgacaacggcctacttcttcacattcaccgagcaatgcatgcagttattgatagacagaaaaatcatggtatgcatttccgtgtattagctaaagcattgcgtatgtctgggggagatcatatccacgctggtacagtagtaggtaagttagaaggggaacgcgaaatgactttaggttttgttgatttattgcgcgatgattttattgaaaaagatcgtgctcgcggtatctttttcactcaggactgggtatccatgccaggtgttataccggtggcttcagggggtattcatgtttggcatatgccagctctgaccgaaatctttggagatgattctgtattgcaatttggtggaggaactttaggacatccttggggtaatgcacctggtgcagcagctaatcgggtggctttagaagcctgtgtacaagctcgtaacgaagggcgcgatcttgctcgtgaaggtaatgaaattatccgatcagcttgcaaatggagtcctgaactagccgcagcttgtgaaatatggaaagcgatcaaattcgagttcgagccggtagataaactagatagctag*

*>Triticum_aestivum*

*atgtcaccacaaacagaaactaaagcaggtgttggatttaaagctggtgttaaagattataaattgacttactacaccccagagtatgaaactaaggatactgatatcttggcagcattccgagtaagtcctcagcctggggttccgcccgaagaagcaggggctgcagtagctgccgaatcttctactggtacatggacaactgtttggactgatggacttaccagtcttgatcgttacaaaggacgatgctatcacatcgagcctgttgctggggaagacagccaatggatctgttatgtagcttatccattagacctatttgaagagggttccgttactaacatgtttacttccattgtaggtaacgtatttggtttcaaagccctacgtgctctacgtttggaggatctacgaattccccctacttgttcaaaaactttccaaggcccgcctcatggtatccaagttgaaagagataagttgaacaagtatggtcgtcctttattgggatgtactattaaaccaaaattgggattatccgcaaaaaattatggtagagcgtgttatgagtgtctacgtggtggacttgattttaccaaagatgatgaaaacgtaaactcacaaccatttatgcgctggagagaccgttttgtcttttgtgccgaagctatttataaatcacaggccgaaaccggtgaaatcaaggggcattacttgaatgcgactgcgggtacatgtgaagaaatgattaagagagctgtatttgcaagagaattaggggttcctattgtaatgcatgactacttaactgggggattcaccgcaaatactactttggctcattattgccgcgacaatggcctacttcttcacattcaccgtgcaatgcatgcagttattgatagacagaaaaatcatggtatgcatttccgtgtattagctaaagcattgcgtatgtctgggggagatcatatccactccggtacagtagtaggtaagttagaaggggaacgcgaaatgactttaggttttgttgatttattgcgcgatgattttattgaaaaagatcgtgctcgcggtatctttttcactcaggactgggtatccatgccaggtgttataccggtagcttcaggtggtattcatgtttggcatatgccagctctgaccgaaatctttggggacgattctgtattacaatttggtggaggaactttaggacatccttggggaaatgcacctggtgcagcagctaatcgagtggctttagaagcctgtgtacaagctcgtaacgaagggcgcgatcttgctcgcgaaggtaatgaaattatccgagcagcttgcaaatggagtcctgaactagccgcagcttgtgaagtatggaaggcgatcaaattcgagttcgagccggtagatactattgataagtag*

*>[Fragaria_ananassa](https://www.ncbi.nlm.nih.gov/Taxonomy/Browser/wwwtax.cgi?id=3747)*

*atgtcaccacaaacagagactaaagcaagtgttggattcaaagctggtgttaaagattataaattgacttattatactccggactatgaaaccaaagatactgatatattggcagcatttcgagtaactcctcaacctggagttccgcctgaggaagcaggggcagcggtagctgcggaatcttctactggtacatggacaactgtatggactgacgggcttaccagtcttgatcgttacaaagggcgatgctaccacatcgaacctgttcctggagaagaaagtcaatttattgcttatgtagcttaccccttagacctttttgaagagggttcggttactaacatgtttacttcgattgtaggtaatgtgtttgggttcaaggccttgcgcgctctacgtctggaggatttacgaatccctactgcttatgttaaaactttccaaggcccgcctcacgggatccaagttgaaagagataaattgaacaagtatggccgccccctattgggatgtactattaaacctaaattggggttatccgctaagaattacggtagagcagtttatgaatgtctccgcggtggacttgattttaccaaagatgatgagaatgttaattcccaaccatttatgcgttggagagaccgtttcttattttgtgccgaagcaatttataaatcgcaggctgaaacaggtgaaatcaaagggcattatttgaatgctactgcaggtacatgcgaagatatgatgaaaagagccgtatttgccagagaattgggagttcctatcgtaatgcatgattacttaacggggggattcactgcaaatactaccttggctcattattgccgagacaacggtctacttcttcacatccaccgtgcaatgcatgctgttattgatagacagaagaatcatggtatgcactttcgtgtactagctaaagcattacgtatgtctggtggagatcatatacacgctggtaccgtagtaggtaagcttgagggggaaagagaaataactttaggctttgttgatttactacgtgatgattttattgaaaaggatcgaagccgcggtatttatttcactcaagattgggtttctttaccgggtgttttaccggtggcttccggcggtattcacgtttggcatatgcctgctctgaccgagatctttggagatgattctgtactacaattcggtggaggaactttaggacacccttggggaaatgcacctggtgccgtagctaatcgagtagctctagaagcatgtgtacaagcccgtaatgagggacgtgatctcgctcgtgagggtaatgacattattcgtgaggcttgtaaatggagtcctgaactagctgctgcttgtgaagtatggaaagagatcaaatttgaattcgaagcaatggatactttgtaa*

*>Selaginella_moellendorffii*

*acgtcaccgcaaacggaaactaaagcaagtgttggatccaaggccggcgtgaaggatcacagattaactcattacactcccgattaccagaccaaagacaccgatattccggcagcatcccgaatgactccgcaacccggagtgcccgccgaagaagcgggagccgcagtagccgcggaatcctccacaggaacgtggactaccgtttggaccgatggactgactaatcttgatcgttataaaggtcggtgctatgatatcgaacccgttccgggggaaaaagatcaatacattgcttatgcagctcatccttcggacctgtctgaggaaggttccgttaccaacatgtccacctccatagtgggtaatgtttttggatccaaggccttacgagccccgcgttcggaagatctgcgaatcccccccgcttattccaagacctccaagggtccaccccatggtatccaagtcgaaagggataaatcgaacaaatatggccgtccctcgctgggatgtactataaaacccaagttgggtctatccgctaaaaactacggcagagcagtccatgaacgtcttcgtggtggactcgatttcaccaaagatgatgagaacgtaaatcctcaaccattcatgcgttggcgagatcgtttcgtattcgtagcggaagctctttataaggctcagtccgaaacaggcgagattaagggtcatcacctgaatgctaccgcgggtacatgcgaagaaatgatgaaaagggcagaattcgctagggaattgggagtgcccatcaccatgcatgaccattcgacaggaggttttaccgcaaatactagtctggcctattattgccgggacaatggtctactcccacacatccatcgcgcaatgcatgctgttattgacagacaaaaaaatcatggtattcatttccgtgtattggccaaagcatcacgcatgtccggtggagatcacattcatggtggtaccgttgtgggtaagcttgagggggaacgccaagtaaccctaggtttcgtggatctgcttcgggatgattatattgataaagaccgaagtcgtggtattcaccctacccaggattgggtatctatgcctggtgtcttgcccgtcgcctccggaggcattcacgtttggcatatgcccgctttgaccgaaatatttggagatgattctgtattacaattcggcgggggcactttgggccacccttgggggaacgcacctggtgcagtagcgaatcgagtcgctttggaagcttgtgtacaagcccgtaacgaaggacgtgatctcgctattgagggtaatgaggttattcgtgaagctagtaagtggagtcctgaactagctgcggcttgcgaggtatggaaagaaatcaagtttgaatttgaaacgattgacactatttga*

*>Ficus_carica*

*atgtcaccacaaacagagactaaagcaagtgttggattcaaagctggtgttaaagattataaattgacttattacactcctgaatatgaagtcaaagatagtgatatcttggcagcatttcgagtaactcctcaacctggagttcctcctgaagaagcaggggcagcggtagctgctgaatcttctactggtacatggacaactgtatggactgacggacttaccagtcttgatcgctacaaaggtcgatgctacaacatcgagcccgttgctggagaagaaaatcaatatattgcttatgtagcttaccctttagacctttttgaagagggttctgttactaatctgtttacttccattgtgggtaatgtatttgggttcaaggccctgcgtgcgctacgtctggaagatttgcgaatccctccttcttatactaaaactttccaaggaccacctcatggtatccaagttgagagagataaattgaacaagtatggccgccccctattgggatgtactattaaacctaaattggggttatccgctaagaattacggtagagcagtttatgaatgtcttcgcggtgggcttgattttaccaaagatgatgagaacgtgaattctcaaccatttatgcgttggagagaccgtttcttattttgtgtcgaagcaatttataaatcacaagctgaaacaggtgaaatcaaaggacattacttgaatgctactgcaggtacatgtgaagaaatgatgaaaagggctgtatttgccagagaattgggagctcctatcgtaatgcatgattacttaacaggaggattcactgcaaatactagcctggctcattattgtcgagataacggtctacttcttcacatccatcgtgcaatgcatgcagttattgatagacagaagaatcatggtatgcactttcgcgtactagctaaagctttacgtctgtctggtggagatcatattcacgcaggtactgtagtaggtaaacttgaaggggaaagagaaatcactttaggatttgttgatttactacgtgatgattttattgaaaaagatcgaagccggggtatttatttcactcaagattgggtttctctaccaggtgttctgcccgtggcttcagggggtattcacgtttggcatatgcctgctttgaccgagatctttggagatgattccgtactacaattcggtggaggaactttaggacatccttggggaaatgcacccggtgccgtagctaatcgagtagctctagaagcatgtgtaaaagctcgtaatgagggacgcgatcttgctcttgagggtaatgaaattattcgtgaggctagtaaatggagtcctgaactagctgctgcttgtgaagtatggaaggaaatcaaatttgaattcgaagcaatggatactttgtaa*

*>Humulus_lupulus*

*atgtcaccacaaacagagactaaagcaagtgttggattcaaagcaggtgttaaagattataaattgacttattacactccggagtatgaaaccaaagatactgatatcttggcagcatttcgagtaactcctcaacctggagttccccctgaagaagctggggctgcggtagctgctgaatcttctactggtacatggacaactgtatggactgacgggcttaccagccttgatcgctacaaaggtcgatgctaccacatcgagcccgttgctggagaagaaaatcaatatattgcttatgtagcttatcccttagacctttttgaagaaggttctgttactaacatgtttacttccattgtgggtaatgtatttgggttcaaagccctgcgcgctctacgtctggaagatttgagaatccctacttcttatactaaaactttccaaggtccgcctcatgggatccaagttgagagagataaattgaacaagtatggccgcccactattgggatgtactattaaacctaaattggggttatccgctaagaattacggtagagcagtttatgaatgtcttcgtggtggacttgattttaccaaagatgatgagaacgtgaattcccaaccatttatgcgttggagagaccgtttcttattttgtgcagaagcaatttataaatcacaggctgaaacaggggaaatcaaaggacattacttgaatgctactgcaggtacatgtgaagaaatgatgaaaagggctgtatttgccagagaattgggagttcctatcgtaatgcatgattacttaacaggaggattcactgcaaatactagcctggctcattattgtcgagataatggtctacttcttcacatccaccgtgcaatgcatgcagttattgatagacaaaagaatcatggtatacactttcgtgtactagctaaagcgttacgtatgtctggtggagatcatatccatgcgggtactgtagtaggtaaacttgaaggggaaagagaaatcactttaggctttgttgatttactacgtgatgattttattgaaaaagatcgaagccgtggtatttatttcactcaagattgggtctctctaccaggtgttctgcccgtggcttcagggggtattcacgtttggcatatgcccgctttgaccgagatctttggagatgattccgtactacaatttggtggaggaactttaggacatccttggggaaatgcacccggtgctgtcgctaatcgagtagctctagaagcatgtgtacaagctcgtaatgagggacgtgatcttgctcgtgagggtaatgaaattattcgtgaggcttgtaaatggagtcctgaactagctgctgcttgtgaagtttggaaggaaatcaaatttgaatttgaagcaatggatacgttgtaa*

*>Arabidopsis_thaliana*

*atgtcaccacaaacagagactaaagcaagtgttgggttcaaagctggtgttaaagagtataaattgacttactatactcctgaatatgaaaccaaggatactgatatcttggcagcattccgagtaactcctcaacctggagttccacctgaagaagcaggggctgcggtagctgctgaatcttctactggtacatggacaactgtgtggaccgatgggcttaccagccttgatcgttacaaaggacgatgctaccacatcgagcccgttccaggagaagaaactcaatttattgcgtatgtagcttatcccttagacctttttgaagaaggttcggttactaacatgtttacctcgattgtgggtaatgtatttgggttcaaagccctggctgctctacgtctagaggatctgcgaatccctcctgcttatactaaaactttccaaggaccacctcatggtatccaagttgaaagagataaattgaacaagtatggacgtcccctattaggatgtactattaaaccaaaattggggttatccgcgaaaaactatggtagagcagtttatgaatgtctacgtggtggacttgattttaccaaagatgatgagaatgtgaactcccaaccatttatgcgttggagagaccgtttcttattttgtgccgaagctatttataaatcacaggctgaaacaggtgaaatcaaagggcattatttgaatgctactgcgggtacatgcgaagaaatgatcaaaagagctgtatttgccagagaattgggagttcctatcgtaatgcatgactacttaacagggggattcaccgcaaatactagtttgtctcattattgccgagataatggcctacttcttcacatccaccgtgcaatgcacgctgttattgatagacagaagaatcatggtatgcacttccgtgtactagctaaagctttacgtctatctggtggagatcatattcacgcgggtacagtagtaggtaaacttgaaggagacagggagtcaactttgggctttgttgatttactgcgcgatgattatgttgaaaaagatcgaagccgcggtatctttttcactcaagattgggtctcactacctggtgttctgcctgtggcttcagggggtattcacgtttggcatatgcctgctttgaccgagatctttggagatgattctgtactacaattcggtggaggaactttaggccacccttggggaaatgcaccgggtgccgtagccaaccgagtagctctggaagcatgtgtacaagctcgtaatgagggacgtgatcttgcagtcgagggtaatgaaattatccgtgaagcttgcaaatggagtcctgaactagctgctgcttgtgaagtatggaaagagatcacatttaacttcccaaccatcgataaattagatggccaagagtag*

*>Cucumis_sativus*

*atgtcaccacaaacagagactaaagcaagtgttggattcaaagctggtgttaaagattataaattgacttattatactcctgaatatgaaaccaaagatactgatatcttggcagcattccgagtaactcctcaaccgggagttccacctgaggaagcaggggccgctgtagctgctgaatcttctactggtacatggacaactgtgtggaccgatgggcttaccagtcttgatcgttacaaaggacgatgctatggaatcgagcctgttgctggagaagaaaatcaatatattgcttatgtagcttatcctctagacctttttgaagaaggttctgttactaacatgtttacttccattgtgggtaatgtatttggattcaaggctctacgtgctctacgtctggaggatttgcgaatccctactgcttatattaaaactttccaaggcccgcctcatggtatccaggttgaaagagataaattgaacaagtatggtcgccctctattgggatgtactattaaaccaaaattgggattatccgctaagaattatggtagagcagtttatgaatgtctacgcggtggacttgattttaccaaagatgatgaaaacgtgaattcccaaccatttatgcgttggagagaccgtttcctattttgtgcggaagctatttttaaatcacaggctgaaacaggtgaaatcaaagacatactgaatgctactgcgggtacatgcgaagaaatgatgaaaagggctatatttgcccgagaattgggagctcctatcgtaatgcatgactacttaacaggtggattcactgcaaatactagcttggctcattattgccgagataatggtctacttcttcacattcaccgtgcaatgcatgctgttattgatagacagaagaatcatggtatgcacttccgtgtactagctaaagcgttacgtatgtctggtggagaccatattcacgctggtaccgtagtaggtaaacttgaaggggaaagagaaatcactttaggctttgttgatttactacgtgatgattttgttgaaaaagaccgaagccgcggtatttatttcactcaagattgggtctctttaccaggtgttctgccagtggcttccggtggtattcacgtttggcatatgcctgctctaaccgagatttttggagatgattctgtactacaattcggcggaggaactttggggcacccttggggtaatgcacctggtgccgtagctaaccgagtagctctagaagcatgtgtacaagctcgtaatgagggacgtgatcttgctcgtgagggtaatgaaattatccgtgaggctagtaaatggagtcctgaactagtctgcgtgacttggaggaagtatggtaaggagatcaaatttgaatttgaggcaatggatactttgtaa*

*>Phaseolus_vulgaris*

*atgtcaccacaaacagagactaaagcaagtgttgggttcaaagctggtgttaaagattataaattgacttattatactcctgactatgaaaccaaagatactgatatcttggcagcattccgagtaactcctcaacctggagttccacctgaagaagcaggtgccgcggtagccgctgaatcttctactggtacatggacaactgtgtggaccgatgggcttaccagtcttgatcgttacaaaggacgatgctatcacatcgaacctgttgctggggaagaaaatcaatttattgcttacgtagcttatcccttagacctttttgaagaaggttctgttactaacatgtttacttctattgtcggtaatgtatttgggttcaaggcactgcgtgctctacgtctggaggatttgcgaatcccaaccgcttatattaaaactttccaaggtccgcctcatggcatccaagttgagagagataaattgaacaagtatggtcgtcccctattaggatgtactattaaacctaaattggggttatccgctaagaattatggtagagcagtttatgaatgtcttcgtggaggacttgattttaccaaagatgatgaaaatgtgaattcccaaccatttatgcgttggagagaccgtttcttattttgtgctgaagcgatttataaagcacaggctgaaacaggtgaaatcaaagggcattacttgaatgcaactgcgggtacatgtgaagaaatgataaaaagagctgtatttgccagagaattaggcgttcctatcgtaatgcatgattatttaacagggggattcactgcaaatactagcttggctcattattgccgggataatggtctacttcttcatatacatcgtgcaatgcatgcagttatcgacagacaaaagaatcatggtatgcactttcgtgtcctagctaaagcattacgtttgtctggtggagatcatgttcactccggtaccgtagtaggtaaacttgaaggggaaagagaaatcactttaggttttgttgacttattgcgtgatgattttattgaaaaagatcgaagtcgtggtatttatttcactcaggattgggtttctctaccaggtgttctgccggttgcttcgggaggtattcacgtttggcatatgcctgctctgaccgaaatctttggagatgactccgtacttcaatttggcggaggaactttaggacacccttggggaaatgcaccaggtgctgtagctaatcgagtagctcttgaagcatgtgtgcaggctcgaaatgaaggacgtgatcttgctcgtgaaggtaatgaaattatccgtgaggctagcaaatggagtcctgaattagctgctgcttgcgaagtatggaaggagatcaaatttgaattcgaagcaatggatactttggattaa*

*>Petunia_hybrida*

*atgtcaccacaaacagagactaaagcaagtgttggattcaaagctggtgttaaagagtacaaattgacttattatactcctgagtaccaaaccaaggatactgatatattggcagcattccgagtaactcctcaacctggagttccacctgaagaagcaggggccgcggtagctgccgaatcttctactggtacatggacaactgtatggaccgatggacttaccagccttgatcgttacaaagggcgatgctaccgcatcgagcgtgttattggagaaaaagatcaatttattgcttatgtagcttaccctttagacctttttgaagaaggttctgttaccaacatgtttacttccattgtaggtaacgtatttgggttcaaagcccttcgcgctctacgtctggaagatctgcgaatccctcctgcttatgttaaaactttccaagggccgcctcatggtatccaagttgaaagagataaattgaacaagtatggtcgtcccctactgggatgtactattaaacctaaattggggttatctgctaaaaactacggtagagctgtttatgaatgtcttcgcggtggacttgattttaccaaagatgatgagaacgtgaactcacaaccatttatgcgttggagagaccgtttcttgttttgtgccgaagcaatttataaatcacaggctgaaacaggtgaaatcaaagggcattacttgaatgctactgcaggtacatgcgaagaaatgatgaaaagagctatatttgctagagaattgggcgttccgatcgtaatgcatgactacttaaccgggggattcaccgcaaatactagcttggctcattattgccgagataatggtctacttcttcacatccaccgtgcaatgcatgcggttattgatagacagaagaatcatggtatccacttccgcgtattagcaaaagcattacgtatgtctggtggagatcatattcactctggtaccgtagtaggtaaacttgaaggtgaaagagacatcactttgggctttgttgatttactgcgtgatgattttgttgaacaagatagaagtcgcggtatttatttcactcaagattgggtctctttaccaggtgttctacccgtggcttcaggaggtattcacgtttggcatatgcctgctctgaccgagatctttggggatgattccgtactacagttcggtggaggaactttaggacacccttggggtaatgcgccaggtgccgtagctaaccgagtagctctagaagcatgtgtacaagctcgtaatgaaggacgtgatcttgctcaagaaggtaatgagattattcgtgaggcttgcaaatggagcccggaactagctgctgcttgtgaggtatggaaagagatccgatttaattttgcagcagtcgacgttttggataagtag*

*>Helianthus_annuus*

*atgtcaccacaaacagagactaaagcaagtgttggattcaaagctggtgttaaagattataaattgacttattatactcctgaatatgaaaccaaggatactgatatcttggcagcatttcgagtaactcctcaacctggagttccgcctgaagaagcaggggccgcagtagctgccgaatcttctactggtacatggacaactgtatggaccgatggacttaccagccttgaccgttacaaaggccgatgctatggacttgagcctgttcctggagaagacaatcaatttattgcttatgtagcgtacccattagacctttttgaagaaggttctgttactaacatgtttacttccattgtaggtaatgtatttgggttcaaagccctgcgtgctctacgtctggaagatttgcgaatcccgactgcgtatgttaaaactttcgacggtccgcctcacggtatccaagttgaaagagataaattgaacaagtatggtcgtcccctgttgggatgtactattaaaccgaaattggggttatccgctaaaaactacggtagagcttgttatgaatgtcttcgtggtggccttgattttactaaagatgatgagaacgtgaactcccaaccatttatgcgttggagagaccgtttcttattttgtgccgaagctatttataaagcacaagctgaaacaggtgaaatcaaagggcattacttgaatgctactgcgggtaattgcgaagatatgatgaaaagggctgtatttgctagagaattgggagttcctatcgtaatgcatgactacctaacaggtggattcactgcaaatactagcttgtctcagtattgccgagataatggtctacttcttcacatccaccgcgcaatgcatgcggttattgatagacagaagaatcatggtatgcacttccgtgtactagctaaagcgttacgtatgtccggtggagatcacattcattccggtaccgtagtaggtaaacttgaaggggaaagagaaatcactttgggctttgttgatttactgcgtgatgattttattgaaaaagatagaagtcgcggtatttatttcacccaagattgggtctctctaccaggtgttctgcctgtagcttcggggggtattcacgtttggcatatgcctgctctaaccgagatctttggggatgattccgtactacagttcggtggaggaactttagggcacccttggggtaatgcacctggtgccgtagctaaccgagtagctctagaagcatgtgtacaagctcgtaatgagggacgcgatcttgctactgagggtaatgaaattatccgtgaggctaccaaatggagtcctgaactagctgctgcttgtgaagtatggaaggagatcaaatttgagttccaggcaatggatactttggatacggataaggataaagataagaagagataa*

*>Nicotiana_tabacum*

*atgtcaccacaaacagagactaaagcaagtgttggattcaaagctggtgttaaagagtacaaattgacttattatactcctgagtaccaaaccaaggatactgatatattggcagcattccgagtaactcctcaacctggagttccacctgaagaagcaggggccgcggtagctgccgaatcttctactggtacatggacaactgtatggaccgatggacttaccagccttgatcgttacaaagggcgatgctaccgcatcgagcgtgttgttggagaaaaagatcaatatattgcttatgtagcttaccctttagacctttttgaagaaggttctgttaccaacatgtttacttccattgtaggtaacgtatttgggttcaaagccctgcgcgctctacgtctggaagatctgcgaatccctcctgcttatgttaaaactttccaaggtccgcctcatgggatccaagttgaaagagataaattgaacaagtatggtcgtcccctgttgggatgtactattaaacctaaattggggttatctgctaaaaactacggtagagctgtttatgaatgtcttcgcggtggacttgattttaccaaagatgatgagaacgtgaactcacaaccatttatgcgttggagagatcgtttcttattttgtgccgaagcactttataaagcacaggctgaaacaggtgaaatcaaagggcattacttgaatgctactgcaggtacatgcgaagaaatgatcaaaagagctgtatttgctagagaattgggcgttccgatcgtaatgcatgactacttaacggggggattcaccgcaaatactagcttggctcattattgccgagataatggtctacttcttcacatccaccgtgcaatgcatgcggttattgatagacagaagaatcatggtatccacttccgggtattagcaaaagcgttacgtatgtctggtggagatcatattcactctggtaccgtagtaggtaaacttgaaggtgaaagagacataactttgggctttgttgatttactgcgtgatgattttgttgaacaagatcgaagtcgcggtatttatttcactcaagattgggtctctttaccaggtgttctacccgtggcttcaggaggtattcacgtttggcatatgcctgctctgaccgagatctttggggatgattccgtactacagttcggtggaggaactttaggacatccttggggtaatgcgccaggtgccgtagctaatcgagtagctctagaagcatgtgtaaaagctcgtaatgaaggacgtgatcttgctcaggaaggtaatgaaattattcgcgaggcttgcaaatggagcccggaactagctgctgcttgtgaagtatggaaagagatcgtatttaattttgcagcagtggacgttttggataagtaa*

*>Hordeum_vulgare*

*atgtcaccacaaacagaaactaaagcaggtgttggatttcaagctggtgttaaagattataaattgacttactacaccccagagtatgaaactaaggatactgatatcttggcagcattccgagtaagtcctcagcctggggttccgcccgaagaagcaggggctgcagtagctgccgaatcttctactggtacatggacaactgtttggactgatggacttaccagtcttgatcgttacaaaggacgatgctatcacatcgagcctgttgctggggaagacagccaatggatctgttatgtagcttatccattagacctatttgaggagggttccgttactaacatgtttacttccattgtgggtaacgtatttgggttcaaagccctacgtgctctacgtttggaggatctacgaattccccctacttattcaaaaactttccaaggcccgcctcatggtatccaagttgaaagagataagttgaacaagtatggccgtcctttattgggatgtactattaaaccaaaattgggattatccgcaaaaaattatggtagagcgtgttatgagtgtctacgtggtggacttgattttaccaaagatgatgaaaacgtaaactcacaaccatttatgcgctggagagaccgttttgtcttttgtgccgaagctatttataaatcacaggccgaaaccggtgaaatcaaggggcattacttgaatgcgactgcgggtacatgtgaagaaatgattaagagagctgtatttgcgagagaattaggggttcctattgtaatgcatgactacttaaccgggggattcaccgcaaatactactttggctcactattgccgcgacaatggcttacttcttcacattcaccgtgcaatgcatgcagttattgatagacagaaaaatcatggtatgcatttccgtgtattagctaaagcattgcgtatgtctgggggagatcatatccactccggtacagtagtaggtaagttagaaggggaacgcgaaatgactttaggttttgttgatttattgcgcgatgattttattgaaaaagatcgtgctcgcggtatctttttcactcaggactgggtatccatgccaggtgttataccggtagcttcaggtggtattcatgtttggcatatgccagctctgaccgaaatctttggggacgattctgtattacaatttggtggaggaactttaggacatccttgggggaatgcacctggtgcagcagctaatcgagtggctttagaagcttgtgtacaagctcgtaacgaagggcgtgatcttgctcgcgaaggtaatgaaattatccgagcagcttgcaaatggagtcctgaactagccgcagcttgtgaagtatggaaggcgatcaaattcgagttcgagccggtagatactatcgataagaaggtctaa*

*>Pisum_sativum*

*atgtcaccacaaacagaaacgaaagcaaaggttgggttcaaagctggtgttaaagattataaattgacttattatactcctgactatcaaaccaaagatactgatatcttggcagcattccgagtaactcctcaacctggagttccgcctgaagaagcaggtgcggcggtagctgcagaatcttccactggtacatggacaactgtgtggaccgatggacttacgagcctcgatcgttataaaggacgctgctacgagatcgagcctgttcctggagaagataatcaatttattgcttatgtagcttatcccttagacctttttgaagaaggttctgttactaacatgtttacctccattgtaggtaatgtatttgggttcaaggccttgcgtgctctacgtctggaagatttgcgaatcccttatgcttatgttaaaactttccaaggtcctcctcacggaatccaagttgagagagataaattgaacaagtatggacgtcccctattgggatgtactattaaaccaaaattgggtttatccgctaagaattatggtagagcagtttatgaatgtctccgcgggggacttgattttaccaaagatgatgaaaatgtgaactcccaaccatttatgcgttggagagaccgtttcttattttgtgccgaagcaatttataaatcacaggccgaaacaggtgaaatcaaaggacattatttgaatgctactgcgggtacatgtgaagaaatgctaaaaagagctgtatttgctagagaattgggcgttcctatcgtaatgcatgactacttaacaggtggattcactgcaaatactaccctgtctcactattgccgggataatggtctacttcttcatatccaccgtgcaatgcatgcagttatcgatagacaaaaaaatcatggtatgcactttcgtgtattagctaaagccttacgtttgtctggtggagatcatattcacgctggtactgtagtaggtaaacttgaaggagaaagggagattactttaggttttgttgatttactacgtgatgattatattaaaaaagatagaagtcgcggtatttatttcactcaggattgggtttctttaccaggtgttatccctgttgcttcagggggtattcacgtttggcatatgcctgctctgaccgagatatttggagatgattctgtactccaattcggtggaggaactttaggacacccttggggaaatgcacctggtgccgtagcgaatcgagtagctctggaagcatgtgtacaagctcggaatgagggacgtgatcttgctcgcgagggtaatgcaattatccgtgaagcttgcaaatggagtcctgaattagctgctgcttgtgaagtctggaaggaaatcaaatttgaattcccagcaatggatactttgtaa*

*>Dianthus_caryophyllus*

*atgtcaccacaaacagagactaaagcaagtgttggatttaaagctggtgttaaagattacaaattgacgtattatactcctgagtatgaaaccctggatactgatatcttggcagcattccgagtaagtcctcaacctggagttccacccgaagaagctggggccgcagtagccgccgaatcttctactggtacatggacaactgtatggaccgacggacttaccagtcttgatcgttacaaaggacgttgctaccacatcgagcccgttgctggagaagaaaatcaatatatttgttatgtagcttatcccttagacctttttgaggaaggctctgttactaacatgtttacttccattgtgggtaatgtatttgggttcaaagccttgcgtgctctacgtttggaggatttgcgaatccctgttgcttatgtaaaaactttcctaggcccgcctcacggcatccaagttgagagagataaattgaacaagtatggccgtccactattgggatgcactattaaaccgaaattggggttatccgctaaaaactatggtcgagcagtttatgaatgtcttcgcggtggacttgattttaccaaagatgatgaaaacgtgaactcccaaccatttatgcgttggagagaccgcttcttattttgtgccgaagcaattaataaagcacaggccgaaacaggtgaaattaaagggcattacttgaatgccactgcgggtacatgtgaagaaatgatcaaaagggctgtatttgccagagaattgggagctcctattgtaatgcatgactacttaacagggggattcactgcaaatactagcttggctcattattgccgagataatggtctacttcttcacatccaccgtgcaatgcacgcagttattgatagacagaagaatcatggtatgcacttccgtgtactagctaaagcgttacgtctgtctggtggagatcatattcatgccggtaccgtagtaggtaagcttgaaggggaaagagaaatcaccttaggctttgttgatttactacgtgatgattttactgaaaaagatcgaagtcgcggtatttatttcactcaatcttgggtttctacaccaggtgttctgcccgttgcttcgggaggtattcacgtttggcatatgcccgctctaaccgagatctttggagatgattccgtactacagtttggtggaggaaccctaggacacccttggggaaatgcaccgggtgctgtagcgaatcgagtagctctagaagcatgtgtacaagctcgtaatgaaggacgtgatcttgctcgcgagggtaatactattattcgcgaggcttgcaaatggagtcctgaactagctgctgcttgtgaagtatggaaggaaatcaaatttgaattcgaagcaatggatacaatctaa*

*>Picea_abies*

*atgtcaccaaaaacagagactaaagctagtgtcggatttaaagctggtgttaaagattacagattaacttattatactcctgaatatcagaccaaagatacggatattttggcagcattccgagtaactcctcaaccaggggtgccgcccgaggaagcgggagcagcagtagctgctgaatcttccaccggtacatggaccactgtttggaccgatggacttactagtcttgatcgttacaaggggcgatgctatgacatcgagcccgttgctggagaggaaagtcaatttattgcctttgtagcttaccccttagaccttttcgaagaaggttctgttactaacttgttcacttccattgtaggtaatgtatttggattcaaggccctacgggctctacgtttggaagatttgcggattccccctgcttattccaaaacatttcaaggtccacctcatggtatccaagttgaaagagataaattgaacaaatatggccgtcctttattgggatgtactatcaaaccaaaattgggtctatcggccaagaactatggtagagcagtttacgaatgtctccgtggtggactcgattttaccaaggatgatgagaacgtaaattcccaaccattcatgcgctggagagatcgttttgtcttttgtgcggaagcaatttataaagctcaggctgagacgggtgaaattaagggacattacttgaatgctactgcaggtacatgtgaagaaatgatgaaaagggcagtatttgcaagagaattgggagttcctatcgttatgcatgactatctgacgggaggttttaccgcaaatacttctttggctcattattgccgagacaacggcctacttcttcacattcaccgcgcgatgcatgcagtgattgacagacaaaaaaatcatggtatgcacttccgtgtactggctaaagcattgcgtatgtccggtggagatcatattcacggcggtactgtagtaggtaaacttgaaggggaacgagaaatcactttagggtttgttgatctactgcgtgatgattttatcgaaaaagatcgaagtcgtggtatttacttcactcaagattgggtatctatgccaggtgtcctgcccgtagcttcaggaggtattcacgtttggcatatgcctgctctgaccgagatctttggggatgattccgtactacagtttggtgggggaactttgggacacccttggggaaatgcacctggtgcagtagctaatcgagttgctctagaagcttgtgtacaagctcgtaatgaaggacgtgatcttgctcgtgaaggtaatgaagtgatccgtgaagctagtaaatggagtcctgaactagctgctgcttgtgaaatatggaaggagatcaaatttgaatttgaggcagtagatacaatttga*

*>Narcissus_tazetta_chinensis*

*ggatttaaagctggtgttaaagattacagattgacttattatactcccgattacgaaaccaaagatactgatatcttggcagcattccgagtaactcctcaacccggagttcccgctgaagaagcaggggctgcggtagctgccgaatcttctactggtacatggacaactgtgtggactgatggacttaccagccttgatcgttacaaaggacgatgctaccacattgaggccgttattggggaagaaaatcaatttattgcttatgtagcttatcctttagacctttttgaagaaggttctgttactaacatgtttacttccattgtgggtaatgtatttggtttcaaagccttacgagctctacgtctggaggatctgcgaattccccctgcttattccaaaactttccaagggcccccccatggcatccaatctgaaagagataaattgaacaagtatggtcgtcccctattaggatgtactattaaaccaaaattgggattatccgcaaaaaactacggtagagcggtttatgaatgtctacgcggtgggcttgattttaccaaggatgacgaaaacgtgaactcccaaccttttatgcgttggagagaccgtttcttattttgtgctgaagcaatttataaagcgcaagccgaaacaggtgaaatcaaaggacattacttgaatgcaactgcgggtacatgtgaagaaatgatcaaaagggccgtatttgccagagaattgggagttcctatcgtaatgcatgactacttaactgggggattcactgcaaatactactttggcttattattgccgcgacaacggtctacttcttcacatccaccgcgcaatgcatgcagttattgatagacagaaaaatcatggtatgcattttcgtgtactagccaaagcattacgtatgtctggtggagatcatattcacgccggtacagtagtaggtaaactggaaggggaacgtgagatgactttaggttttgttgatttattacgtgatgattttattgaaaaagaccgaagtcgcggtatttttttcactcaagattgggtttctatgccaggtgttattcccgtagcttccgggggtattcatgtttggcatatgcccgccctaaccgaaatctttggagatgattccgtactacagttcggtggaggaactttaggacacccttggggaaatgcacctggtgcggtagctaatcgggtagctttagaagcgtgtgtacaagctcgtaatgaaggacgtgatcttgctcgtgaaggtaatgaaattatccgcgaagcttgcaaatggagccctgaactagc*

*>Hyacinthus_orientalis*

*taaagcaggtgttggattcaaagctggtgttaaagattacagattgacttattatactcctgattacgaaaccaaagatactgatattttggcagcattccgagtaactcctcaacctggagttcctgctgaagaagcaggggctgcggtagctgccgaatcttctactggtacatggacaactgtgtggaccgatggacttaccaatcttgatcgttacaaaggacgatgctaccacattgaggccgttgttggggaagaaaatcaatttattgcttatgtagcttatcctttagacctttttgaagaaggttctgttactaacatgtttacttccattgtgggtaatgtatttggtttcaaagccctacgagctctacgtctggaggatttgcgaattcctccttcttattccaaaactttccaaggcccgcctcacggcatccaagttgaaagagataaattgaacaagtacggtcgtcccctattgggatgtactattaaaccaaaattgggattatccgcaaaaaactacggcagagcggtttatgagtgtctgcgtggtgggcttgattttaccaaggatgatgaaaacgtgaactcacaaccttttatgcgttggagagaccgtttcttattttgtgctgaagcaatttataaagcacaagccgaaacaggtgaaattaaaggacattacttgaatgcaactgcaggtacatgtgaagaaatgatcaaaagagccgtatttgccagagaattgggagttcctatcgtaatgcatgactacttaactgggggattcactgcaaatacgagtttagctcattattgccgtgataacggtctacttcttcatatccaccgcgcaatgcatgcagttattgatagacagaaaaatcatggtatgcattttcgtgtactagctaaagcattgcgtatgtcnggtggagatcatattcacgctggtacagtagtaggtaaactggaaggggaacgtgagatgactttaggttttgttgatttattacgtgatgattttattgaaaaagaccgaagtcgcggtatttttttcactcaagattgggtttctatgccnggtgttattcccgtggcttcagggggtattcatgtttggcatatgcctgccctaaccgaaatctttggagatgattcngtactacagttcggtggaggaactttaggacacccttggggaaatgcacctggtgcggtagctaatcgngtagctttagaagcgtgtgtacaagctcgtaatgaggggcgtgatcttgctcgtgaaggtaatgaaattattcgcgaggcttgcaaatggagccctgaactagctgctgcttgtgaa*

*>Allium_cepa*

*atgtcaccacaaacagaaactaaagcaagtgttggatttaaagctggtgttaaagattacagattgacttattatactcctgattacgaaaccaaagatactgatatcttagcagcattccgagtaactcctcaacccggagttcccgctgaagaagcaggggctgcggtagccgccgaatcttctactggtacctggacaactgtgtggactgatggacttaccagtcttgatcgttacaaaggacgatgctaccacattgaggccgttgttggggaagaaaatcaatatattgcttatatagcttatcctttagacctttttgaagaaggttctgttactaacatgtttacttccattgtgggtaatgtatttggtttcaaagccctgcgagctctacgtttagaggatctgcgaattccccctgcttattccaaaactttccaaggcccgcctcacggcatccaagttgaaagagataaattgaacaagtatggacgtcccctattgggatgtactattaaaccaaaattgggattatccgcaaaaaactacggtagagcagtttacgaatgtctacgtggtgggcttgattttaccaaggatgatgaaaatgtgaactcccagccttttatgcgttggagagaccgtttcttattttgtgctgaagctatttataaagcgcaagctgaaacaggtgaaatcaaagggcattacttgaatgcaactgcaggtacatgtgaagaaatgatcaaaagggctgtatttgccagagaattgggagttcctatcataatgcatgactacataaccgggggattcactgcaaatactagtttggctcattattgccgagacaatggtctacttcttcacatccaccgcgcaatgcatgcagttattgatagacagaaaaatcatggtatgcattttcgtgtactagctaaagcattacgtatgtctggtggagatcatattcacgctggtacagtagtaggtaaactggaaggggaacgtgagatgactttaggttttgttgatttattacgtgatgattttattgaaaaagaccgaagtcgcggtatttttttcactcaagattgggtttctatgccaggtgttattcccgtggcttccgggggtattcatgtttggcatatgcctgccctaactgaaatctttggagatgattccgtcctacagttcggtggaggaactttagggcacccttggggaaatgcacctggtgcggtagctaatcgagtagctttagaagcgtgtgtacaagctcgtaatgaaggacgtgatcttgctcgtgaaggtaatgagattatccgcgaagcttgcaaatggagtcccgaacttgcggctgcttgtgaagtatggaaagagatcaaattcgagttcgaaccagtagataagatagataaacagaaatag*

*>Allium_sativum*

*atgtcaccacaaacagaaactaaagcaagtgttggatttaaagctggtgttaaagattacagattgacttattatactcctgattacgaaaccaaagatactgatatcttagcagcattccgagtaactcctcaacccggagttcccgctgaagaagcaggggctgcggtagccgccgaatcttctactggtacctggacaactgtgtggactgatggacttaccagtcttgatcgttacaaaggacgatgctaccacattgaggccgttattggggaagaaaatcaatttattgcttatatagcttatcctttagacctttttgaagaaggttctgttactaacatgtttacttccattgtgggtaatgtatttggtttcaaagccctgcgagctctacgtttagaggatcttcgaattccccctgcttattctaaaactttccaaggcccgcctcacggcatccaagttgaaagagataaattgaacaagtatggacgtcccctattgggatgtactattaaaccaaaattgggattatccgcaaaaaactacggtagagcagtttacgaatgtctacgtggtgggcttgattttaccaaggatgatgaaaatgtgaactcccagccttttatgcgttggagagaccgtttcttattttgtgctgaagctatttataaagcgcaagctgaaacaggtgaaatcaaagggcattacttgaatgcaactgcgggtacatgtgaagaaatgatcaaaagggctgtatttgccagagaattgggagttcctatcgtaatgcatgactacataaccgggggattcactgcaaatactagtttggctcattattgccgagacaatggtctacttcttcacatccaccgcgcaatgcatgcagttattgatagacagaaaaatcatggtatgcattttcgtgtactagctaaagcattacgtatgtctggtggagatcatattcacgctggtacagtagtaggtaaactggaaggggaacgtgagatgactttaggttttgttgatttattacgtgatgattttattgaaaaagaccgaagtcgcggtatttttttcactcaagattgggtttctatgccaggtgttattcccgtggcttccgggggtattcatgtttggcatatgcctgccctaactgaaatctttggagatgattccgtcctacagttcggtggaggaactttagggcacccttggggaaatgcacctggtgcggtagctaatcgagtagctttagaagcatgtgtacaagctcgtaatgaaggacgtgatcttgctcgtgaaggtaatgagattatccgcgaagcttgcaaatggagtcccgaacttgcggctgcttgtgaagtatggaaagagatcaaattcgagttcgaaccagtagataagatagataaacagaaatag*

*>Raphanus_sativus*

*atgtcaccacaaacagagactaaagcaagtgttggattcaaagctggtgttaaagagtataaattgaattattatactcctgaatatgaaaccaaggatactgatatcttggcagcattccgagtaactcctcaacccggagttccacctgaagaagcaggggctgcggtagctgctgaatcttctactggtacatggacaactgtgtggaccgatgggcttaccagccttgaccgttacaaaggccgatgctaccacatcgagcccgttccaggagaagaaactcaatttattgcgtatgtagcttaccccttagacctttttgaagaagggtctgttactaacatgtttacctcaattgtgggtaacgtatttgggttcaaagccctggctgctctacgtctagaggatctgcgaatccctccggcttatactaaaactttccagggaccacctcatggtatccaagttgaaagagataaattgaacaagtatggacgtcccctattaggatgtactattaaacctaagttggggttatccgcgaagaactatggtagagcagtttatgaatgtctacgtggtggacttgattttaccaaagatgatgagaatgtgaactctcaaccatttatgcgttggagagaccgtttcttattttgtgccgaagctatttataaatcacaggctgaaacaggtgaaatcaaaggacattatttgaatgctactgcgggtacatgcgaagaaatgatgaaaagagctatatttgccagagaattgggagttcctatcgtaatgcatgactacttaacagggggattcaccgcaaatactagtttggctcattattgccgagataatggcctacttcttcacatccaccgtgcaatgcacgctgttattgatagacagaagaatcatggtatgcacttccgtgtactagctaaagctttacgtctatcgggtggagatcatgttcacgcgggtacagtagtaggtaaacttgaaggagacagggagtcaactttgggctttgttgatttactgcgcgatgattatgttgaaaaagaccgaagccgtggtatctttttcactcaagattgggtctcactaccaggtgttctacctgtggcttcagggggtattcacgtttggcatatgcctgctttgactgagatctttggagatgattccgtactacaatttggtggcggaactttaggccacccttggggaaatgcaccgggtgccgtagctaaccgagtagctctagaagcatgtgtacaagctcgtaatgagggacgtgatcttgcagtcgagggtaatgaaattatccgtgaggcttgcaaatggagtcctgaactagctgctgcttgtgaagtatggaaggagatcacatttaacttcccaaccatcgataaattagatggccaagactag*

*>Saintpaulia_ionantha*

*cgggtgttaaagagtacaaattgacttattatactcctgaatacgaaaccaaagatacggatatcttggcggcattccgagtaactcctcaacctggagttccgcctgaagaagcaggggcggcggtagctgccgaatcttctactggtacatggacaactgtgtggaccgatgggcttaccagccttgatcgttacaaagggcgatgctaccacatcgatcccgttcctggagaaacagatcaatatatctgttatgtagcttaccctttagacctttttgaagaaggttctgttactaacatgtttacttccattgtaggtaatgtatttggattcaaagcccttcgtgctctacgtctggaagatctgcgaatccctactgcttatgttaaaactttccaaggcccgcctcatgggatccaagttgaaagagataaattgaacaagtatggtcgtcccctgttgggatgtactattaaaccaaaactggggttatctgctaaaaactacggtagagcggtttatgaatgtcttcgcggcggacttgattttaccaaagatgacgagaacgtgaactcccagccatttatgcgttggagagatcgtttcttattttgtgccgaagctatttataaatcacaggctgaaacaggtgaaatcaaagggcattacttgaatgctactgcgggtacatgcgaagaaatgatgaaaagggctatatttgctagagaattgggagttcctatcgtaatgcatgactacttaacaggaggattcactgcaaatactagtttggctcattattgccgagataatggcctacttcttcacattcaccgtgcaatgcatgcagttattgatagacagaagaatcatggtatacatttccgtgtattagctaaagcgctacgtatgtctggtggagatcacattcacgctggtaccgtagtaggtaaacttgaaggagaaagagacatcactttgggctttgttgatttactgcgtgatgattttattgcaaaagatcgaagtcgcggtatttatttcactcaagattgggtctctctaccgggtgttattccggtggcttcagggggtattcacgtttggcatatgcctgctctgaccgagatctttggggatgattctgtactacagttcggtggaggaactttaggacacccttggggtaatgcgccaggtgccgtagctaaccgagtagctgtagaagcatgtgtacaagctcgtaatgaaggacgtgatcttgctgctgagggtaacgcaattatccgtgaggctagcaaatggagtcctgaactagctgccgcttgtgagatatggaaagagatcaaatttgaatttcaagcggtggatactttggatgaggaaaaaaagtaacaaacaggtaattactctccgttctcttaattgaatttcaattaaactcggcccaatcttttactaaaag*

*>Socratea_exorrhiza*

*atgtcaccacaaacagaaactaaagcaagtgttggatttaaagctggtgttaaagattacaaattgacttattatactcctgactacgaaaccaaagatactgatatcttggcagcattccgagtaactcctcaacccggagttccgcctgaggaagcaggggcagcggtagctgccgaatcttctactggtacatggacaactgtgtggactgatggacttaccagtcttgatcgttacaaaggacgatgctaccacatcgaaaccgttgtcggggaggaaaatcaatatattgcttatgtagcttatcctttagacctttttgaagaaggttctgttactaacatgtttacttccattgtgggtaatgtatttggtttcaaagccctacgagctctacgtctggaggatctgcgaattcccacttcttattccaaaactttccaaggcccgcctcatggcatccaagttgaaagagataagttgaacaagtatggtcgtcctctattgggatgcactattaaaccaaaattgggattatccgcaaagaactacggtagagcggtttatgaatgtctacgcggtggacttgattttaccaaggatgatgaaaacgtgaactcacaaccatttatgcgttggagagaccgtttcttattttgtgccgaagcactttttaaagcgcaggccgaaacgggtgaaatcaaaggacattacttgaatgctactgcgggtacatgtgaagaaatgatcaaaagggccgtatttgccagagaattgggagttcctatcgtaatgcatgactacttaactgggggattcactgcaaatactagcttggctcattattgccgcgataacggcctacttcttcacatccatcgcgcaatgcatgcagttatcgatagacagaaaaatcatggtatgcattttcgtgtactagctaaagcattacgtatgtctggtggagatcatattcacgcgggtacagtagtgggtaaactggaaggggaacgtgagatgactttgggttttgttgatttattacgtgatgattttattgaaaaagaccgaagtcgcggtatcttttttactcaagattgggtctctatgccaggtgttatccccgtggcttcagggggtattcatgtttggcatatgcctgccctgaccgaaatctttggggatgattccgtactacagtttggcggaggaactttaggacacccttggggaaatgcacccggtgcagtagctaatcgggtggctttagaagcgtgtgtacaagctcgtaatgaaggacgtgatcttgctcgtgaaggtaatgaaattatccgtgaagctagcaaatggagccctgaactagctgccgcttgcgaaatatggaaggagatcaaattcgaattccaaccagtggatacgctggataaatga*

*>Rhizophora_mangle*

*atgtcaccacaaacagagactaaagcaagtgttggatttaaggctggtgttaaagattataaattgacttattatactcctgactatgaaaccaaagatactgatatcttggcagcattccgagtaactcctcaacctggagttccgcctgaggaagcaggagctgcggtagctgctgaatcttctactggtacatggacaaccgtgtggaccgatgggcttaccagtcttgatcgttataaaggacgatgctaccacatcgaggcagttgctggagaagaaaatcaatatattgcttatgtagcttaccccttagacctttttgaagaaggttctgttactaatatgtttacttcgattgtaggtaatgtatttgggttcaaagccctacgcgctctacgtctggaggatttgcgaattcctacttcttatattaaaactttccaaggcccacctcatggcatccaagttgagagagataaattgaacaagtatggtcgccccctattgggctgtactattaaacctaaattggggttatccgctaagaattacggtagagcggtttatgaatgtctccgtggtggacttgattttaccaaagatgatgagaacgtgaattcacaaccatttatgcgctggagagatcgtttcttattttgtgccgaagcaatttataaagcacaggcggaaactggtgaaatcaaagggcattattttaatgctactgcaggtacatgtgaagaaatgatgaaaagggctgtatgtgccagagaattgggagttcctatcgtaatgcacgactacttaacagggggattcactgcaaatactagcttggctcattattgtcgagataatggtttacttcttcacattcatcgcgcaatgcatgcagttattgatagacagaagaatcatggtatgcactttcgtgtactagctaaggcattacgtctgtctggcggggaccatattcatgctggtaccgtagtaggtaaacttgaaggggaaagagacatcactttgggctttgttgatctacttcgtgatgattatattgaaaaagatcgaagccgtggtatttatttcactcaagattgggtttctttaccaggtgttctgcccgtggcttcagggggtattcatgtttggcatatgcctgctctgaccgagatctttggagatgattccgtactacaattcggtggaggaactttaggacacccttggggaaatgcaccaggtgctgtagctaatcgagtagctctagaagcatgtgtccaagctcgtaatgagggacgtgatcttgctcgtgagggtaatgaaattatccgtgaggctagcaaatggagtccggaactagctgctgcttgtgaagtatggaaagagattaaatttgaattcccagcaatggatactttgtaa*

*>Larix_laricina*

*gtcggattcaaagctggtgttaaagattacagattaacttattatactcctggatatcagaccaaagatacggatatcttggcagcattccgagtaactcctcaacctggggtgccacccgaggaagcgggagcagcagtagctgctgaatcttccaccggtacatggaccactgtttggaccgatggacttactagtcttgatcgttacaagggacgatgctatgacatcgaggccgttcctggagaggagagtcaatttattgcctatgtagcttaccccttagaccttttcgaagaaggttctgttactaacttgttcacttccattgtaggtaatgtatttggattcaaggccctacgggctctacgtttggaagatttgcggatcccccctgcttattccaaaacttttcaaggtccacctcatggtatccaagtcgaaagggataaattgaacaaatatggccgtcctttattgggatgtactatcaaaccaaaattgggtctatcggctaagaactatggtagagcagtttacgaatgtctccgtggtggacttgattttaccaaggatgatgagaacgtaaattcccaaccattcatgcgctggagagatcgttttgtcttttgtgcggaagcactttataaggctcaggctgagacgggtgaaattaagggacattacttgaatgctactgcaggtacatgtgaagaaatgatgaaaagggcaatatttgcaagagaattgggagttcctatcgttatgcatgactatctgacgggaggttttactgcaaatacttctttggctcattattgccgagacaacggcctacttcttcacattcaccgcgcgatgcatgcagttattgacagacaaagaaatcatggcatgcatttccgtgtactggctaaagcattgcgtatgtccggtggagatcatattcacgccggtactgtagtaggtaaacttgaaggggaacgagacgtcactttagggtttgttgatctactgcgtgatgattttattgaaaaagatcgaagtcgtggtatttacttcactcaagactgggtatctatgccaggtgttctgcccgtagcttcaggaggtattcacgtttggcatatgcctgctctgaccgagatctttggggatgattccgtactacagtttggtgggggaactttggggcacccttggggaaatgcgcctggtgcagtagctaatcgggttgctctagaagcttgtgtacaagctcgtaatgaaggacgtgatcttgctcgtgaaggtaatgaagtgatccgtgaagctactaaa*

*>Mangifera_indica*

*atgtcaccacaaacagagactaaagcaagtgttggattcaaagccggcgttaaagactataaattgacttattatactcctgactatataaccaaagatactgatatcttggcagcattccgagtaactcctcaacctggagttccacccgaggaagcaggggctgcggtagctgcggaatcttctactggtacatggacaactgtgtggaccgatgggcttaccagccttgatcgttacaaaggacgatgctacaacattgagcccgttgctggagaagaaaatcaatatatatgttatgtagcttaccctttagacctttttgaagaaggttctgttactaacatgtttacttccattgtgggtaatgtatttgggttcaaagccctgcgcgctctacgtctagaggatctacgaatccctaccgcgtatataaaaactttccaaggaccaccgcatgggatccaagttgagagagataaattgaacaagtatggccgtcccctattgggatgtactattaaaccgaaattaggtttatccgctaagaactacggtagagctgtttatgaatgtctacgtggtggacttgactttaccaaagacgatgagaacgtgaactcccaaccatttatgcgttggagagaccgtttcctattttgtgcggaagctctttttaaagcgcaggctgaaacaggtgaaattaaaggtcattacttgaatgctactgcaggtacatgcgaagaaatgattaaaagggctatatttgcaagagagttgggagctcctatcgtaatgcatgactacttaacagggggattcaccgcaaatactagcttggctcattattgccgagataatggtctacttcttcacatccatcgtgcaatgcatgcagttattgatagacagaagaatcatggtatgcactttcgtgtactagctaaagctttacgtatgtctggtggagatcatattcacgccggtacagtagtaggtaaacttgaaggggaaagagacataactttgggctttgttgatttactacgtgatgattttattgaaaaagatcgaagccgtggtatttatttcactcaagattgggtctctttaccaggtgttctgcccgtggcttcagggggtattcacgtttggcatatgcctgctttgaccgagatctttggagatgattccgtactacaattcggtgggggaactttaggacacccttggggaaatgcgccgggcgccgtagctaatcgagtagctctagaagcatgtgtacaagctcgtaatgaaggacgcgatcttgctcgcgagggtaatgaaattatccgtgaggctagcaaatggagtcctgaactggctgctgcttgtgaagtatggaaggagatcaaatttgaattcgaagcaatggatactttgtaa*

*>Hedera_helix*

*atgtcaccacaaacagagactaaagcaagtgttggattcaaagctggtgttaaagattacagattgacttattatactcctgactatgaaaccaaagatactgacatcttggcagcattccgagtaactcctcaacctggagttccacctgaagaagcaggggccgcggtagctgccgaatcttctactggtacatggacaactgtgtggaccgatggacttaccagccttgatcgttacaaagggcgatgctacggaatcgagcccgttgctggagaagaaagtcaatttattgcttatgtagcttacccattagacctttttgaagaaggttctgttactaacatgtttacttccattgtaggtaatgtatttgggttcaaagccctgcgtgctctacgtctggaagatctgcgaatccctgttgcttatgttaaaactttccaaggcccgcctcatggcatccaagttgagagagataaattgaacaagtatggtcgtcccctgttgggatgtactattaaacctaaattggggttatctgctaaaaactacggtagagcggtttatgaatgtctccgtggtggacttgattttaccaaagacgatgagaacgtgaactcccaaccatttatgcgctggagagatcgtttcgtattttgtgccgaagcactttataaagcgcaggctgaaacaggtgaaatcaaagggcattacttgaatgctactgcgggtacatgcgaagaaatgatgaaaagggctgtatttgccagagaattgggagttcctatcgtaatgcatgattacttaacagggggattcactgcaaatactaccttggctcattattgccgagataatggcctacttcttcacatccaccgcgcaatgcatgcagttattgatagacagaagaatcatggtatgcactttcgtgtactagctaaagggttacgtatgtctggtggagatcatattcactccggtaccgtagtaggtaaacttgaaggggaaagagacatcactttgggctttgttgatttactgcgtgatgatttcattgaaaaagatcgaagtcgcggtatttatttcacccaagattgggtctctctaccaggtgttctgcccgtggcttcggggggtattcacgtttggcatatgcctgctctgaccgagatctttggggatgattccgtactacagttcggtggaggaactttaggacacccttggggaaatgcacccggtgccgtagctaatcgagtagctctagaagcatgtgtacaagctcgtaatgagggacgtgatcttgctcgtgaaggtaatgaaattatccgtgaggctgctaaatggagccctgaactagctgctgcttgtgaggtatggaaggagatcaaatttgaatttgccgcaatggatgttttgtaa*

*>Opuntia_quimilo*

*atgtcaccacaaacagaaactaaagcaagtgttggatttaaagcaggtgttaaagattacaaattgacttattatactcccgaatatcaaccccaggataccgatatcttggcagcatttcgagtaacccctcaacctggagttccgtcagaagaagcaggagccgcagtagctgccgaatcttctactggtacatggacaactgtatggaccgacggacttaccagtcttgatcgttacaaaggacgatgctaccacatcgatgccgttcctggagaagacaatcaatatatttgttatgtagcttacccattagacctttttgaagaaggttctgttactaatatgtttacttccattgtgggtaatgtatttgggttcaaggccctgcgtgctctacgtttggaggatttgcgaatccctgttgcttatataaaaactttccaaggcccgcctcacggtatccaagttgagagagataaattgaacaagtatggccgtcctctactgggatgcactattaagccgaaattggggttatccgctaaaaactatggtcgagcagtttatgaatgtcttcgcggtggacttgattttaccaaagatgacgaaaacgtgaactcccagccatttatgcgttggagagaccgtttcttattttgtgccgaagcaatttataaagcacaggccgaaacaggtgaaatcaaagggcattacttgaatgctaccgcaggtacatgcgaagaaatgataaaaagggctgtatttgccagagaattgggtgttcctatcgtaatgcatgactacttaacaggtggattcactgcaaatactagcttggctcattattgccgagataacggtctactccttcacatccatcgtgcaatgcacgcagttattgatagacagaagaatcatggtatgcacttccgtgtactagctaaagcgttacgtctgtctggtggagatcatattcatgctggtaccgtagtaggtaagcttgaaggggaaagagatatcactttaggctttgttgatttactacgtgatgattatactgaaatagacgcaaatcgcggtatttatttcactcaatcttgggtttccacaccaggtgttctgcccgttgcttcgggaggtattcacgtttggcatatgcccgctctaaccgagatctttggggatgattccgtactacagttcggtggaggaactttaggacacccttgggggaatgcaccgggtgctgtagcgaatcgagtagctctagaagcatgtgtacaagctcgtaatgagggacgtgatcttgctcgcgaaggtgctacaattattcgcgatgctagcaaatggagtcctgaactagccgctgcttgtgaggtatggaaagaaataaaatttgagttcccggcagtggatactttggataaaaagaaaggataa*

*>Agave_americana*

*atgtcaccacaaacagagactaaagcaagtgttggatttaaagctggtgttaaagattacagattgacttattatactcctgattacgaaaccaaagatactgatatcttggcagcattccgagtaactcctcaacctggggttccccctgaagaggcaggggctgcggtagctgcggaatcttctactggtacatggacaactgtgtggactgatggacttaccagtcttgatcgttacaaaggacgatgctaccacattgaggccgttgttggggaagaaaatcaatacattgcttatgtagcttatcctttagacctttttgaagaaggttctgttactaacatgtttacttccattgtaggtaatgtatttggtttcaaagccctacgagctctacgtctggaggatctgcgaattccccctgcttattccaaaactttccaaggcccgcctcacggcatccaagttgaaagagataaattgaacaaatatggtcgtcccctattgggatgtactattaaaccaaaattgggattatccgcaaaaaactacggtagagcggtttatgaatgtctacgcggtgggcttgattttaccaaggatgatgaaaacgtgaactcacagccttttatgcgctggagagaccgtttcttattttgtgctgaagcaatttataaagcacaagccgaaacaggcgaaatcaaaggacattacttgaatgcaactgcaggtacatgtgaagaaatgatcaaaagggccgtatttgccagagaattgggagttcctatcgtaatgcatgactacttaactgggggattcactgcaaatactagtttggctcattattgccgcgacaacggtttacttcttcatatccaccgcgcaatgcatgcagttattgatagacagaaaaatcatggtatgcattttcgtgtactagctaaagcattacgtatgtctggtggagatcatattcacgctggtacagtagtaggtaaactggaaggggaacgtgagatgactttaggttttgttgatttattacgtgatgattttattgaaaaagaccgaagtcgcggtatttttttcactcaagattgggtttctatgccaggtgttattcccgtggcttcagggggtattcatgtttggcatatgcctgccctgaccgaaatctttggagatgattccgtactacagttcggtggaggaactttaggacacccttggggaaatgcacctggtgcggtcgctaatagggtagctttagaagcatgtgtacaagctcgtaatgagggacgcgatcttgctcgcgaaggtaatgagattatccgcgaagcttgcaaatggagccctgaactagccgctgcttgtgaagtatggaaagagatcaaattcgaattcgaaccagtagataagatagataaacagaaagcataa*
